# Emerging Capabilities of Nonclassical Noncovalent Interactions and Asymmetric Catalysis in Stereoselective Glycosylations and Carbohydrate Functionalizations

**DOI:** 10.1021/acs.accounts.5c00289

**Published:** 2025-06-12

**Authors:** Amal Tom Sebastian, Charles C. J. Loh

**Affiliations:** 8797 University College Dublin, UCD School of Chemistry, Belfield, Dublin 4, Ireland

## Abstract

Deriving inspiration from frontier catalytic paradigms has emerged as a major force to tackle long-standing stereoselectivity issues in carbohydrate synthesis. In particular, there is a strong momentum in the harnessing of ***nonclassical σ-hole based noncovalent interactions (NCIs) in chemical glycosylations*** and the use of ***asymmetric catalysis to surmount the formidable site-selectivity challenge*** in the functionalization of carbohydrate polyols.

In this Account, we describe our pioneering contributions to advancing these two major directions. First, we introduce our early work whereby halogen bonding (XB) interactions could be harnessed catalytically on sugars. We realized that the polyoxygenated motifs embedded in different regions of the carbohydrate scaffold offered multiple anchoring points where the XB-catalyst could iteratively interact via halogen**···**O interactions. As a consequence, a counterintuitive multistage XB-activation concept was discovered. In our XB-catalyzed strain-release glycosylation, we intriguingly observed substantial elevation of anomeric selectivity over a wide array of glycosyl substrates as compared with thiourea catalysis. In XB-catalyzed 2-deoxyglycosylations, the multistaged XB-activation phenomena was also operative. Apart from the broader tolerance of glycosyl donors/acceptors compared to thiourea catalysis, we demonstrated the halogen tunability concept, where a halogen swap on the catalyst enabled tolerance of sensitive pentose-based donors.

Next, we discovered that the two σ-holes per chalcogen property of phosphonochalcogenide (PCH) catalysts imparted unique benefits in glycoside activation. This opened up an unknown bifurcated chalcogen bonding (ChB) activation concept that paved a stereoselective entry into 7-ring sugars through either an internal nucleophilic substitution (S_N_i) type mechanism or an intramolecular aglycone transposition strategy. *C*- and *N*-glycosylations of indoles with glycals were realized through conformational distortion by a network of ChB and π-interactions. The exclusively α-selective *O*- and *S*-iminoglycosylation of iminoglycals was further developed through an unprecedented multistep ChB-activation manifold.

Second, our investigations revealed that multiple stereoselectivity challenges in site-selective carbohydrate functionalizations can be concomitantly tackled by an asymmetric catalytic system. Departing from the classical use of asymmetric catalysis to create chiral centers on achiral substrates, we advanced stereochemical complexity generation on sugars by simultaneously addressing the site-, diastereo-, and enantioselectivity challenges when carbohydrate polyols react with prochiral electrophiles. Additionally, rarely observed dynamic kinetic resolution type glycosylations on reducing sugars were unravelled when chiral Rh­(I) and chiral copper catalytic systems were employed. We also discovered that the multiple stereoselectivity control by chiral Pd/organoboron-catalyzed site-selective functionalization of carbohydrate polyols can be attributed to the vital stereocontrolling role of NCIs such as CH−π interactions and hydrogen bonding.

## Graphical abstract

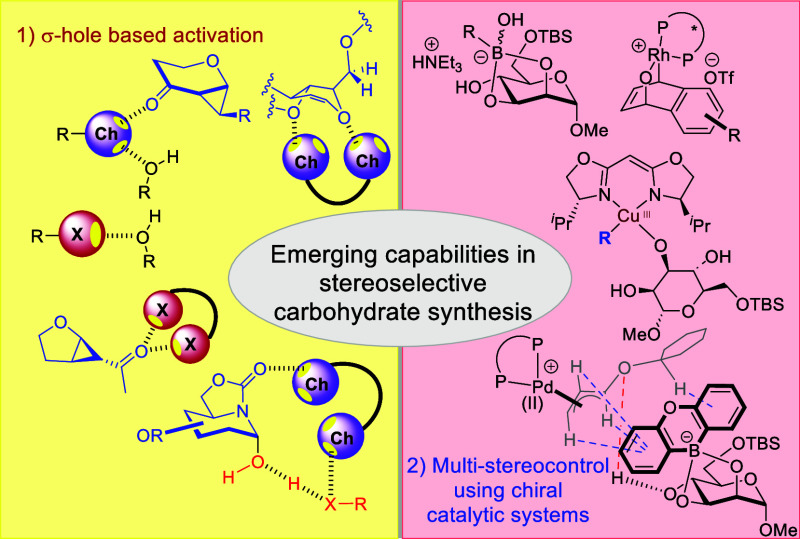

## KEY REFERENCES

- Xu, C.; Loh, C. C. J. A Multistage Halogen Bond Catalyzed Strain-Release Glycosylation unravels New Hedgehog Signaling Inhibitors. *J. Am. Chem. Soc*. **2019**, *141*, 5381–5391.^1^ *This publication laid down the pioneering foundations pertaining to the usage of σ-hole based catalysis as a broad strategy in selective carbohydrate synthesis, and demonstrated stereoselectivity elevation of halogen bonding catalysis over hydrogen bonding-based thiourea catalysis*.
- Ma, W.; Kirchhoff, J-L.; Strohmann, C.; Grabe, B.; Loh, C. C. J. Cooperative Bifurcated Chalcogen Bonding and Hydrogen Bonding as Stereocontrolling Elements for Selective Strain-Release Septanosylation. *J. Am. Chem. Soc*. **2023**, *145*, 26611–26622.^2^ *This research is the seminal chalcogen bonding catalyzed glycosylation strategy in the field of carbohydrate chemistry, documenting for the first time that ChB catalysis can be harnessed in glycosylations. Furthermore, a previously unknown bifurcated ChB mode of activation was unravelled*.
- Bhaskara Rao, V. U.; Wang, C.; Demarque, D. P.; Grassin, C.; Otte, F.; Merten, C.; Strohmann, C.; Loh, C. C. J. A Synergistic Rh­(I)/Organoboron Catalyzed Site Selective Carbohydrate Functionalization that involves Multiple Stereocontrol. *Nat. Chem*. **2023**, *15*, 424–435.^3^ *This research demonstrates a rare carbohydrate functionalization instance where multiple stereoselectivity challenges such as enantio-, diastereo-, site-selectivity can be concomitantly tackled by an asymmetric synergistic catalytic system. Dynamic kinetic resolution type glycosylation that generated 1,2-cis glycosides was unravelled*.
- Guo, H.; Kirchhoff, J.-L.; Strohmann, C.; Grabe, B.; Loh, C. C. J. Asymmetric Pd/Organoboron-Catalyzed Site-Selective Carbohydrate Functionalization with Alkoxyallenes Involving Noncovalent Stereocontrol. *Angew. Chem., Int. Ed.* **2024**, *63*, e202400912.^4^ *This research unveiled the existence of stereocontrolling NCIs (CH−π and hydrogen bonding) in the stereoselectivity determining step of transition-metal catalysis to steer site-selective carbohydrate functionalization. It demonstrates how carbohydrate site selectivity is governed by both catalyst and substrate control*.

## 1 Introduction

Stereoselective carbohydrate synthesis is undoubtedly one of the most fascinating naturally occurring reactions in the eyes of an organic chemist. Besides the well-recognized fact that carbohydrates are essential in almost all aspects of molecular biological phenomena,^5^ sugars are endowed with unparalleled molecular complexity. A glance at the structural features of the ubiquitous d-glucose unit reveals an elegant combination of architectural sophistication (Figure 1A): (i) d-glucose is homochiral; (ii) d-glucose presents several functionality equivalent hydroxyl groups that differ in their stereochemical environment; (iii) these hydroxyl groups further comprise primary and secondary alcohols and hemiacetals; and (iv) the anomeric center exists as a mixture of α- and β-anomers in its reducing or anomerically unprotected form. These four features intriguingly reveal the long-standing fundamental challenges^6^ in the precise stereocontrolled chemical synthesis of natural and unnatural sugars.

**Figure 1:**
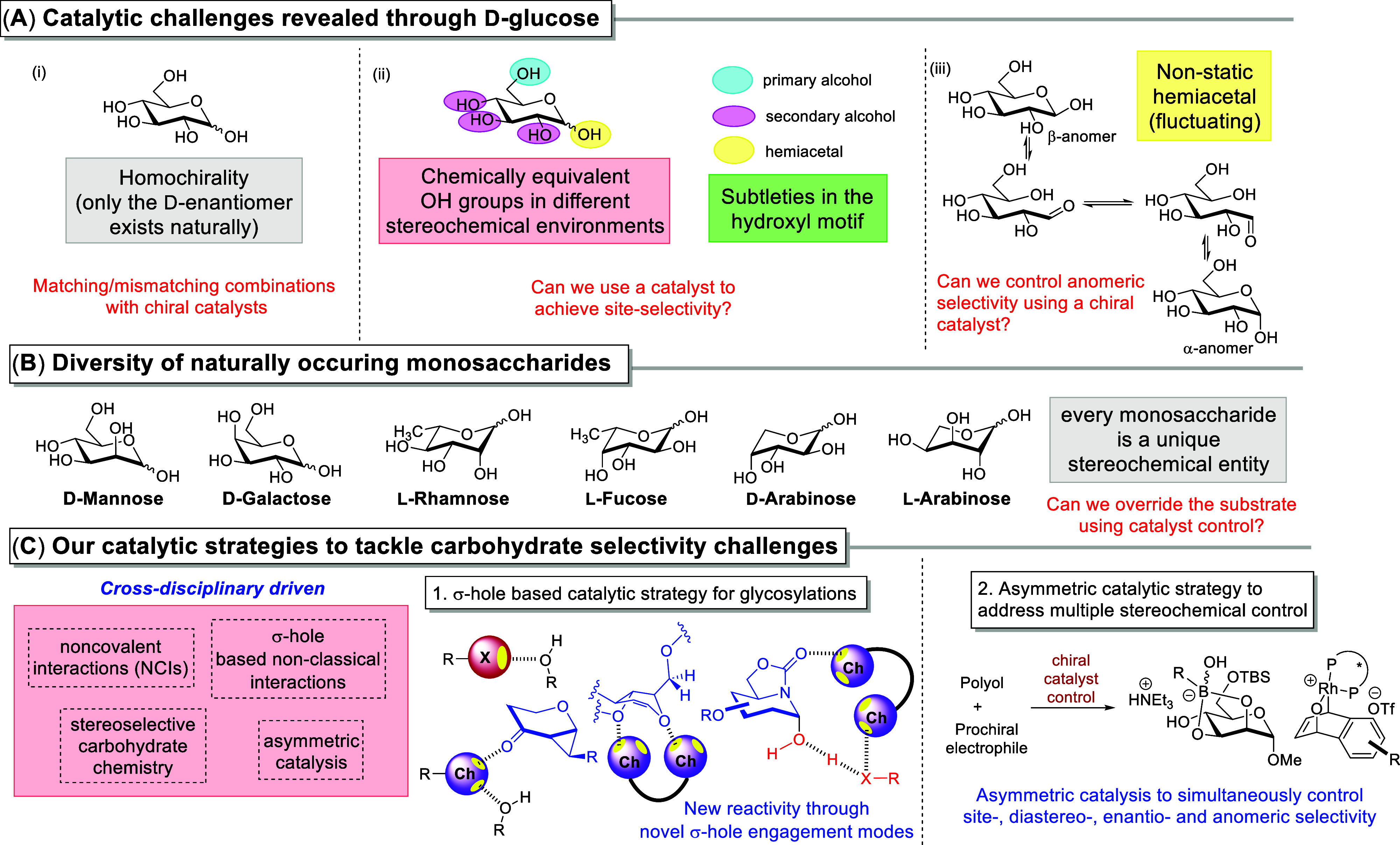
Summary of our two strategic lines to tackle different stereoselectivity challenges in carbohydrate synthesis.

First, the polyol scaffold brings out the synthetic challenge of site-selectivity or regioselectivity^6–12^ that has to be tackled in order to achieve targeted functionalization. Second, the fluctuating conversion between the α- and β-anomers at equilibrium reflects the yet-to-be universally addressed problem of anomeric selectivity/diastereoselectivity.^13^ Third, the homochirality of sugars indicates that any envisioned asymmetric catalytic strategy would require a consideration of matching/mismatching combinations between the chiral catalyst and the chiral sugar.^14^ Furthermore, there is a diversity of naturally occurring monosaccharide building blocks (Figure 1B) which elevates the challenge of catalyst control since each sugar represents a unique stereochemical environment.

The main-stream approach in the past decades had been focused on substrate design,^15^ protecting group modifications^16^ and mechanistic investigation^17^ from the perspective of the sugar substrates. Therefore, a substantial body of valuable knowledge exists on this front. Much less is however known in how vital synthetic concepts of noncovalent interactions (NCIs)^6–8,18^ and asymmetric catalysis could be useful handles to address carbohydrate-based stereoselectivity issues.

The first question we set our sights on in 2017 was whether nonclassical σ-hole-based noncovalent interactions^19,20^ could be in any way beneficial to carbohydrate chemistry (Figure 1C). Back then, such highly directional σ-hole based interactions, which include halogen bonding (XB)^21,22^ and chalcogen bonding (ChB)^23–25^ were only in their infant stages of catalytic exploration. Their existence, however, was already a long-discussed phenomena within the crystallographic and computational circles. σ-hole-based interactions can be attributed to a blend of factors which include the electrostatics, polarization, dispersion, and charge transfer.^22,24^ This results in a remarkable noncovalent phenomenon, whereby “soft” halogens or chalcogens could confer geometrically defined electropositive site(s) or σ-holes for Lewis acidic activation due to electronic anisotropy. Despite the fact that Lewis acids like TMSOTf and Brønsted acids are well established as important stoichiometric promoters in glycosyl donor activation, there exists a widespread assumption of functional equivalency with modern means of catalytic activation.

We were therefore interested to scrutinize this assumption in light of emerging knowledge from asymmetric hydrogen bonding (HB) donor based noncovalent catalysis and in early proof-of-principles of using XB donors in glycosylations. On the one hand, remarkable advances were made by Jacobsen in advancing chiral *bis*-thiourea catalyst controlled glycosylations that confers stereocontrol through a network of HB interactions.^26,27^ On the other hand, there were sporadic instances of XB-activation of glycosyl halides by Codée and Huber,^28^ and the use of XB donors to elevate the acidity of thiourea by Takemoto.^29^ Despite these examples, there was no clear indication of what opportunities σ-hole-based NCIs could offer to the glycosylation problem.

Since oxygenated functional groups such as alcohols, carbonyls, acetals, and ethers are often encountered in carbohydrates, we were driven by the plausibility of harnessing reversible σ-hole interactions catalytically to engage different variants of glycosyl substrates. We reasoned that such an approach could profoundly alter the reactivity and stereoselectivity profiles of glycosylations through new mechanistic pathways. Notably, this concept was also lately picked up by Wang and co-workers for achieving glycosylations using tellurium-based catalysts.^30^

Second, we were interested in exploiting the arsenal of asymmetric catalysis to address complexity generation in site-selective carbohydrate functionalizations (Figure 1C).^14^ Despite the strength of asymmetric catalysis in modern synthesis, these overwhelmingly involved transformations that uses *mono*-functionalized nucleophiles.^31^ The scenario is complicated when multiple chemically equivalent nucleophiles situated in close vicinity within a chiral environment are presented to the electrophile. Furthermore, the use of a prochiral electrophile would demand a higher level of catalytic control. Site-selective functionalizations of carbohydrate polyols with a prochiral electrophile constitutes one such unique transformation,^9,11,12,32^ where the asymmetric catalyst has to address the following stereoselectivity issues: (i) the site-selectivity problem to discriminate the multiple hydroxyl groups; (ii) the enantioselectivity problem in the desymmetrization of a prochiral electrophile; (iii) the diastereoselectivity problem in the bond forming step that creates the new stereogenic center; (iv) the chemoselectivity problem if more than one electrophilic sites are available. Sporadic examples of using chiral transition-metal catalysis on prochiral electrophiles were only known from the groups of Niu in propargylations,^33^ and the group of Tang in chiral Rh­(II) catalyzed alkyations.^34^ The application of reducing sugars on prochiral electrophiles would further elevate the challenge as the nonstatic hemiacetal at the anomeric carbon needs to undergo a dynamic kinetic resolution. The catalytic control hinges on the interception of a chiral catalytic system on the rapidly equilibrating α- or β-anomer to favor a single stereochemical outcome. This domain is relatively underexplored, and only rare instances from the Tang group,^35^ and achiral variants from the Takemoto group^36^ were available in the literature.

This Account will hence summarize our contributions in the two rapidly expanding directions. We will first describe our first generational development of exclusively XB-catalyzed glycosylations prior to 2020, and the associated new mechanistic insights. Next, we showcase our second generational debut featuring ChB-catalyzed glycosylations after 2023. We highlight unexpected mechanistic profiles and depict unique advantages that were not replicable by alternative catalytic modes. Significantly, we brought forth evidence that XB and ChB differ in the activation of glycosyl substrates. Finally, we will detail our contributions in the identification of useful asymmetric Rh­(I), Pd(0), and copper radical catalytic systems in the site-selective alkylation of carbohydrate polyols with prochiral electrophiles. Significantly, these strategies contributed two rare literature instances of dynamic kinetic resolution-type glycosylations by functionalizing the anomeric hemiacetal of reducing sugars with a prochiral electrophile.

## 2 THE σ-HOLE BASED CATALYTIC STRATEGY IN CHEMICAL GLYCOSYLATIONS

The early roots of σ-hole based activation capabilities in carbohydrate chemistry could be traced to an unselective proof-of-principle *O*-glycosylation demonstration by Codée and Huber,^28^ as well as the co-catalytic role of XB to elevate the Brønsted acidity of thiourea via soft halogen-sulfur interactions for glycofunctionalizations by Takemoto.^29^ Notably, Niu lately used stoichiometric amounts of XB-promoter for radical glycosylations.^37^ However, the development of catalytic σ-hole-based interactions as a broadly applicable glycosylation concept has not yet been convincingly demonstrated.

We were hence driven by the alluring possibility that reversible XB interactions between an XB donor catalyst and the numerous oxygens presented on a saccharide scaffold could have far-ranging consequences in glycosyl activation. Particularly, this could set a precedent for noncovalent catalytic engagements/disengagements with both glycosyl donor and acceptor substrates, along with downstream intermediates, and define new mechanistic pathways in the process.

### 2.1 Emergence of the Halogen Bonding (XB)-Catalyzed Glycosylation Approach

In 2018, we embarked on our pioneering adventure^1^ to learn how σ-hole-based catalysis could differentiate itself through exploration of emerging XB-catalytic concepts on cyclopropanated glycosyl donors.^38^ We uncovered that Huber’s *bis*-benzoimidazolium^39^ XB donor **A** gave almost quantitative yields of the *O*-glycoside product with excellent anomeric selectivity. Intriguing was the fact that, in all examples, the XB-catalyzed congener consistently yielded superior anomeric selectivities of >20:1, which out-performed many instances in our previously developed thiourea-catalyzed method (Scheme 1).^40^ Furthermore, a palette of biologically interesting *N*-acceptors such as indolines, benzotriazoles, adenine, and guanine derivatives were amenable with preservation of excellent anomeric selectivity.

**Scheme 1:**
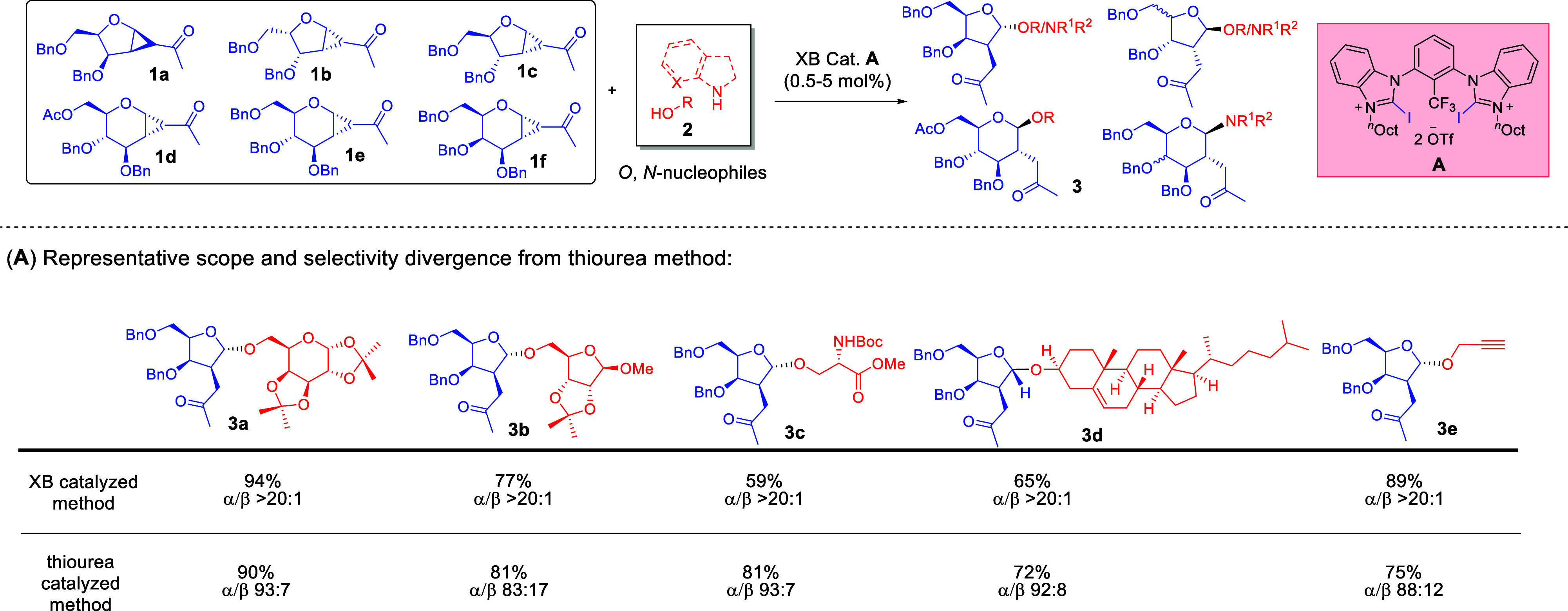
Development of Our Seminal XB-Catalyzed Strain-Release Glycosylation

Through the use of NMR titrations, we obtained evidence to support that XB interactions, such as halogen–hydroxyl interactions and halogen–carbonyl interactions, were operative. We further conducted in-situ NMR monitoring, as well as sequential in-situ NMR monitoring experiments. This led to the elucidation of three crucial intermediates by which a transient charged intermediate **6**, a neutral bicyclic intermediate **4** and a ketal **10** were generated. By separating **4** from the catalyst through filtration over deactivated silica (Scheme 2A), we determined through a sequential addition of a glycosyl acceptor that a downstream glycosylation will not occur in the absence of an XB catalyst. This led us to the postulate that a multistep XB activation manifold (Scheme 2B) that comprises catalytic engagement events of the catalyst with glycosyl donor, glycosyl acceptor in upstream and downstream elementary steps is operative.

**Scheme 2:**
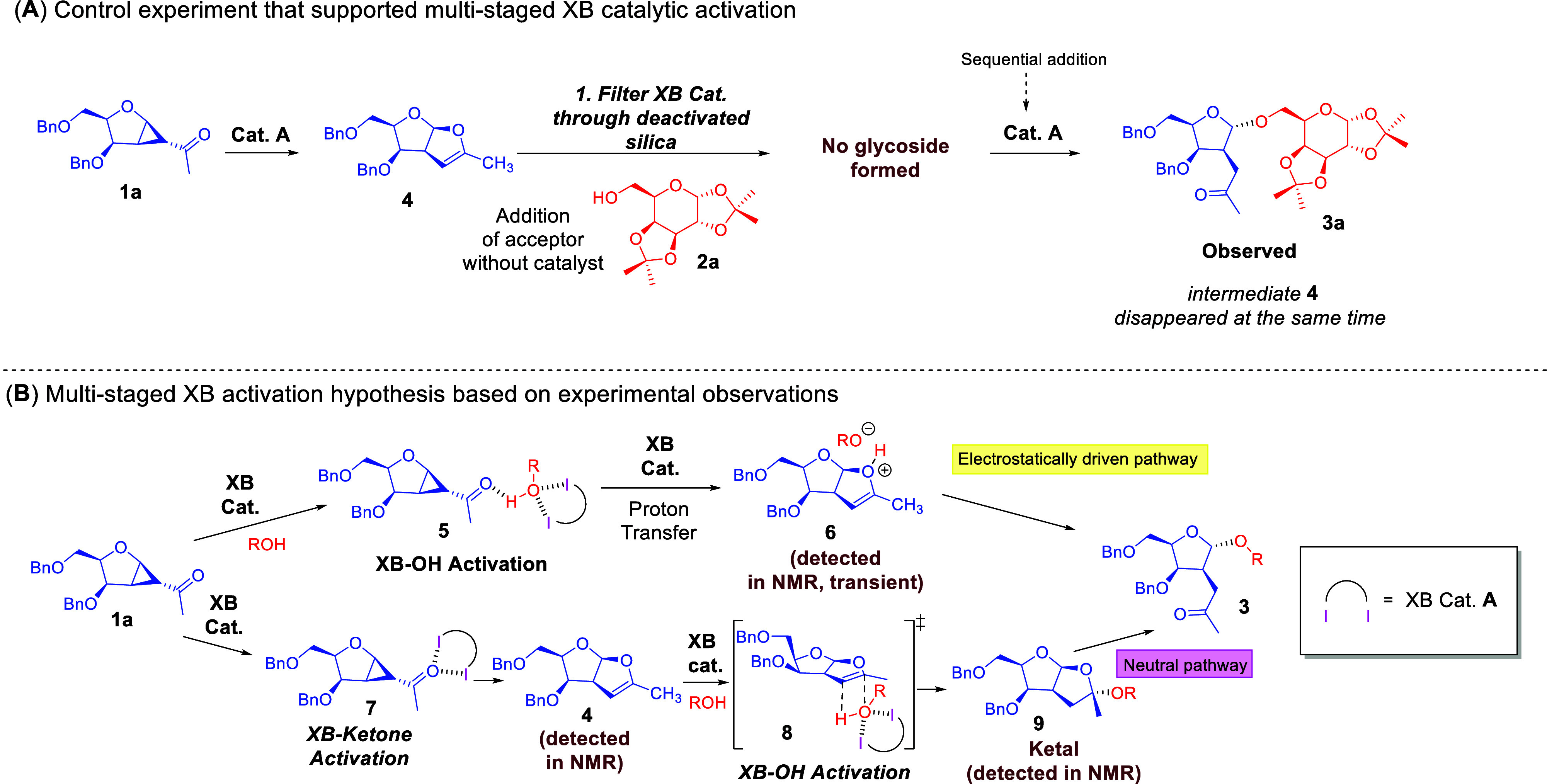
(A) Control Experiment That Unveiled the Multistaged Nature of XB Catalytic Activation; (B) Proposed Mechanism Based on NMR Titration and In-Situ ^1^H NMR Monitoring^a^

a Adapted from ref 1. Copyright 2019, American Chemical Society, Washington, DC.

Seeking to expand the multistaged XB strategy into a more general concept, we identified the 2-deoxyglycosylation platform from glycals. While we were aware that there were precedents in HB-based thiourea-catalyzed 2-deoxygalactosylations,^41^ we were rather puzzled by substrate limitations that restricted to the usage of the more reactive galactal, along with strict requirements of moisture-free protocols. As such, we were keen to pursue the question of whether the unique XB-catalytic reactivity could bring about differentiated 2-deoxyglycosylation profiles under mild reaction conditions.

Interestingly, we demonstrated that XB catalysis using catalyst **B** tolerates a substantially broader array of glycals that include commonly encountered hexoses as well as pentoses (Scheme 3).^42^ Benchmarking experiments against thiourea^41^- or thiouracil^43^-catalyzed protocols (Scheme 4) known from the literature revealed the broader versatility of XB catalysis to accommodate a wider array of glycosyl donors and acceptors. We further discovered a “*halogen swap*” strategy that involved the modification of an *iodo*-XB-donor **B** with the bromo-congener **Br**–**B** (Scheme 5). This introduced a tunability facet of σ-hole catalysis that opens new options for tailoring the σ-hole size of the halogen toward the reactivity of the glycosyl donor. We determined that sensitive glycosyl donors such as the ribal substrate **11c** benefit from the “*halogen swap*” strategy, as the reactivity attenuated *bromo*-XB variant led to product formation in cases where **B** led to product decomposition.

**Scheme 3:**
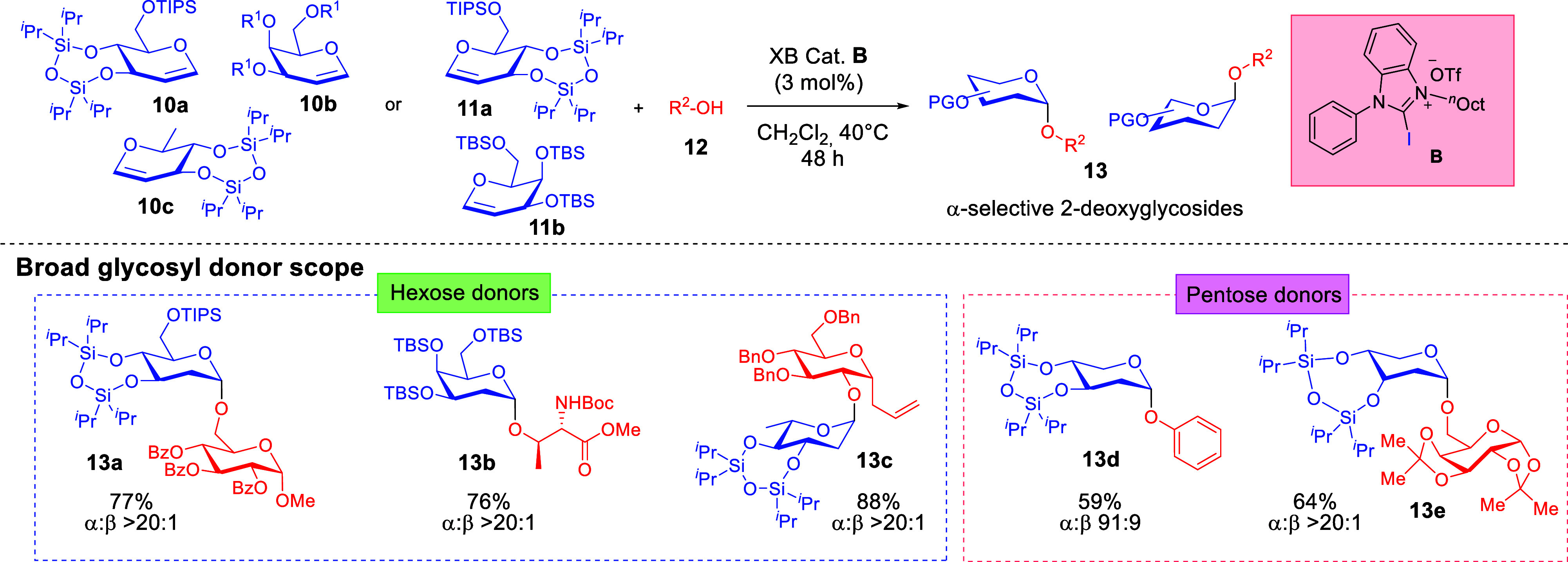
Development of a XB-Catalyzed 2-Deoxyglycosylation That Accommodates a Broad Array of Glycosyl Donors and Acceptors^a^

a Adapted from ref 42, under an open-access CC-BY 4.0 License. Copyright 2020, Springer Nature.

**Scheme 4:**
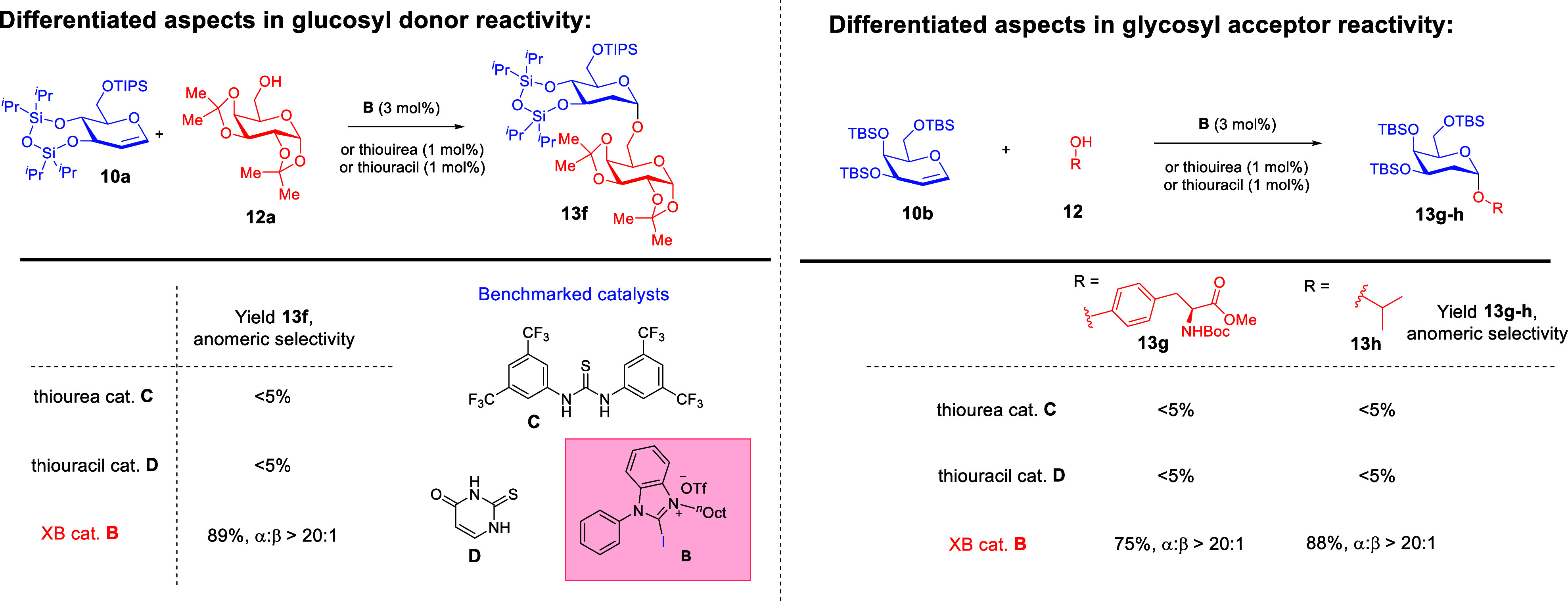
Differing Reactivity of the XB Donor Catalyst versus Classical Thiourea/Thiouracil Catalysis^a^

a Adapted from ref 42, under an open-access CC-BY 4.0 License. Copyright 2020, Springer Nature.

**Scheme 5:**
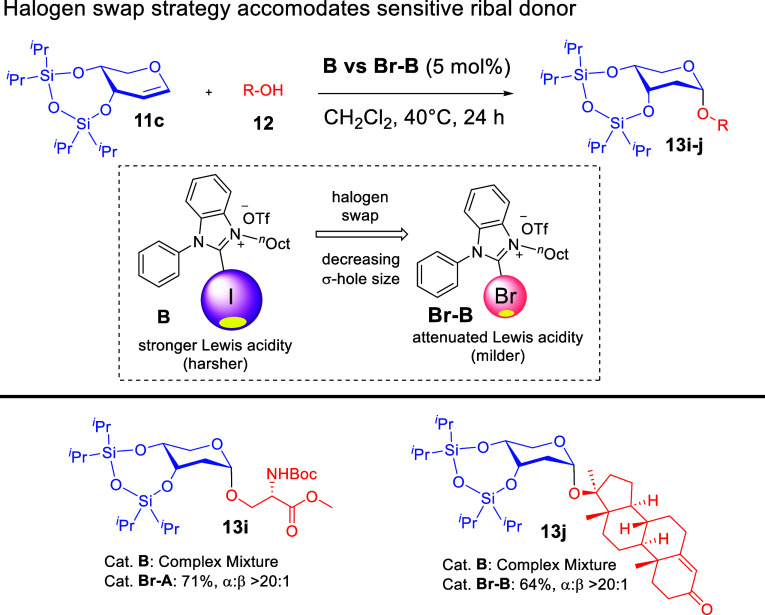
Tunability of XB Catalysis Exemplified by the “*Halogen Swap*” Strategy on Ribals^a^

a Adapted from ref 42, under an open-access CC-BY 4.0 License. Copyright 2020, Springer Nature.

Mechanistic investigations through NMR titrations and control experiments yielded intriguing insights (Scheme 6). We observed that XB donor **B** again displayed multistep σ-hole-based catalytic reactivity in the mechanism. Our working mechanistic proposal involved a host of halogen-oxygen interactions between the catalyst and the acceptor’s hydroxyl group, between the catalyst and the oxyanion after alcohol deprotonation and finally between the catalyst and the glycosidic bond’s oxygen. Based on unusually high but reproducible kinetic isotopic effect (KIE) data, we discovered that the XB-activation of the alcohol led to a hydrogen tunneling effect, which is responsible for a sigmoidal kinetic profile.

**Scheme 6:**
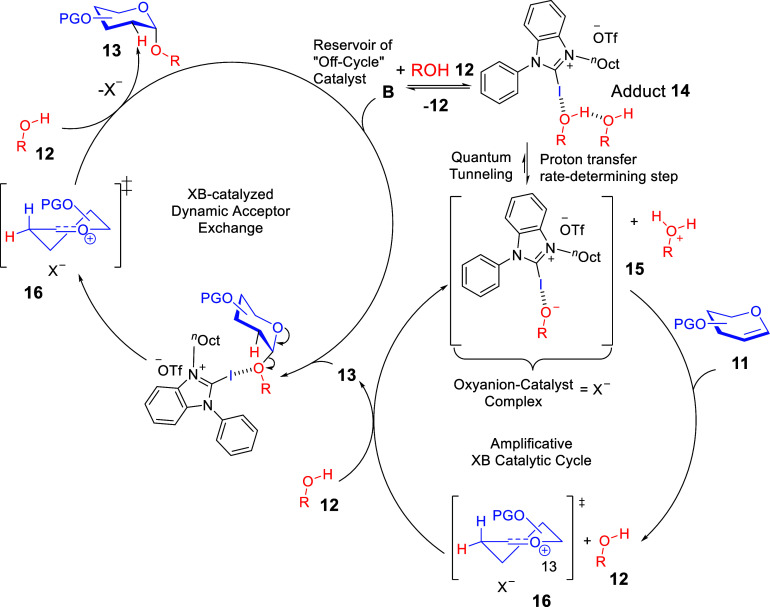
Participation of XB Activation in Multiple Elementary Steps of 2-Deoxyglycosylations^a^

a Adapted from ref 42, under an open-access CC-BY 4.0 License. Copyright 2020, Springer Nature.

### 2.2 Debut of the Chalcogen Bonding (ChB)-Catalyzed Glycosylation Approach

The success of our foray into XB catalysis led us to the broader question of whether σ-hole interactions beyond XB would result in different reactivity or selectivity outcomes. The emergence of a modular class of phosphonochalcogenide (PCH) catalysts^25^ by Wang in 2020 was instrumental in addressing this question. Appreciating the geometric differences of the electrophilic axes and the availability of two σ-holes on chalcogens as compared to the one σ-hole that resides in a 180° disposition in halogens (Scheme 7),^24^ we were curious if these subtleties could be translated to supramolecular catalytic benefits by differentiated engagement of halogen/chalcogens on spatially defined oxygenated moieties on carbohydrates.

**Scheme 7:**
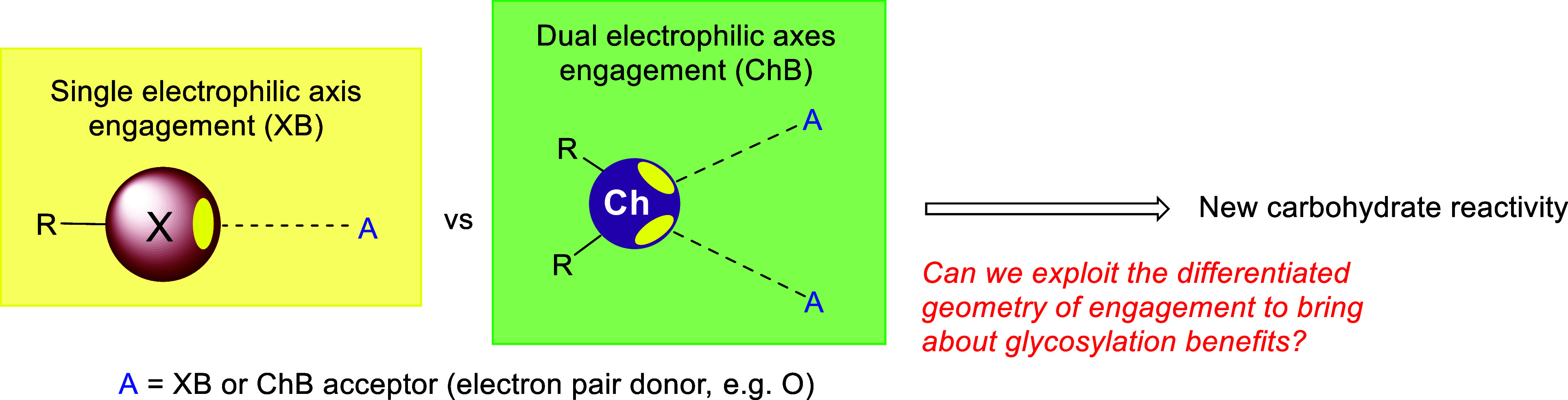
Exploring the Differences in Electrophilic Axes of XB and ChB Catalysts to Bring about Differentiated Glycosylation Outcomes

Our first breakthrough in a second-generation ChB-catalyzed glycosylation occurred around early 2022 while we were studying new routes toward a biologically interesting class of 7-ring sugar known as septanosides,^44,45^ which contain a privileged oxepane core found in marine natural products^46^ and bioactive molecules.^47^ Accessing such structures through a thermodynamically favored strain-release glycosylation strategy^1,40^ was attractive as literature methods involved entropically less desirable macrocyclizations.^48^ Surprisingly though was the realization that strain-release ring expansion approaches into septanosides in the 1990s by Hoberg^49–51^ and Nagarajan^52^ were largely underexplored and plagued with substrate-dependent stereoselectivity issues.^49,50^

By evaluating a palette of noncovalent catalysts, we discovered exclusive catalytic activation when strained cyclopropanated donor **17** was reacted with a model galactosyl acceptor at 2 mol % catalyst loading of PCH catalyst **C** (Scheme 8).^2^ A vast range of *O*- and *S*-nucleophiles could be accommodated with consistently excellent α-selectivity. It was however noteworthy that halogen bonding donors and the Schreiner’s thiourea were completely ineffective.

**Scheme 8:**
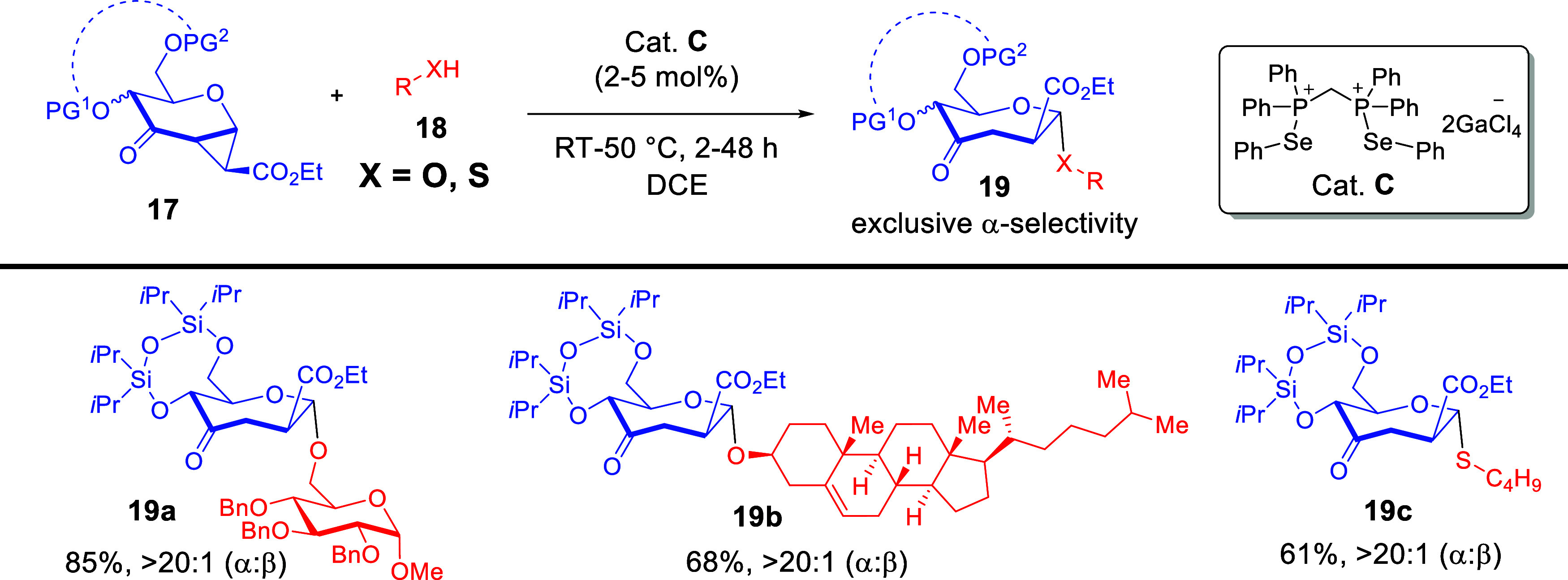
Seminal Report for the Debut of the ChB-Catalyzed Glycosylation^a^

a Adapted from ref 2, under an open-access CC-BY 4.0 License, Copyright 2023, American Chemical Society, Washington, DC.

We delved deeper into the mechanistic workings by using ^77^Se and ^13^C NMR titrations, along with in-situ ^13^C NMR monitoring. Besides observing notable chemical shift perturbations evidencing selenium–carbonyl and selenium-hydroxyl interactions, we detected downfield “shoulder peaks” that suggested the formation of a ternary catalyst-donor–acceptor complex. DFT calculations (Scheme 9A) further revealed the existence of a ternary assembly that is held together by a bifurcated ChB, a conventional ChB and a hydrogen bond. The postulate of a ternary complex was further supported by comparing two- and three-component NMR titrations.

**Scheme 9:**
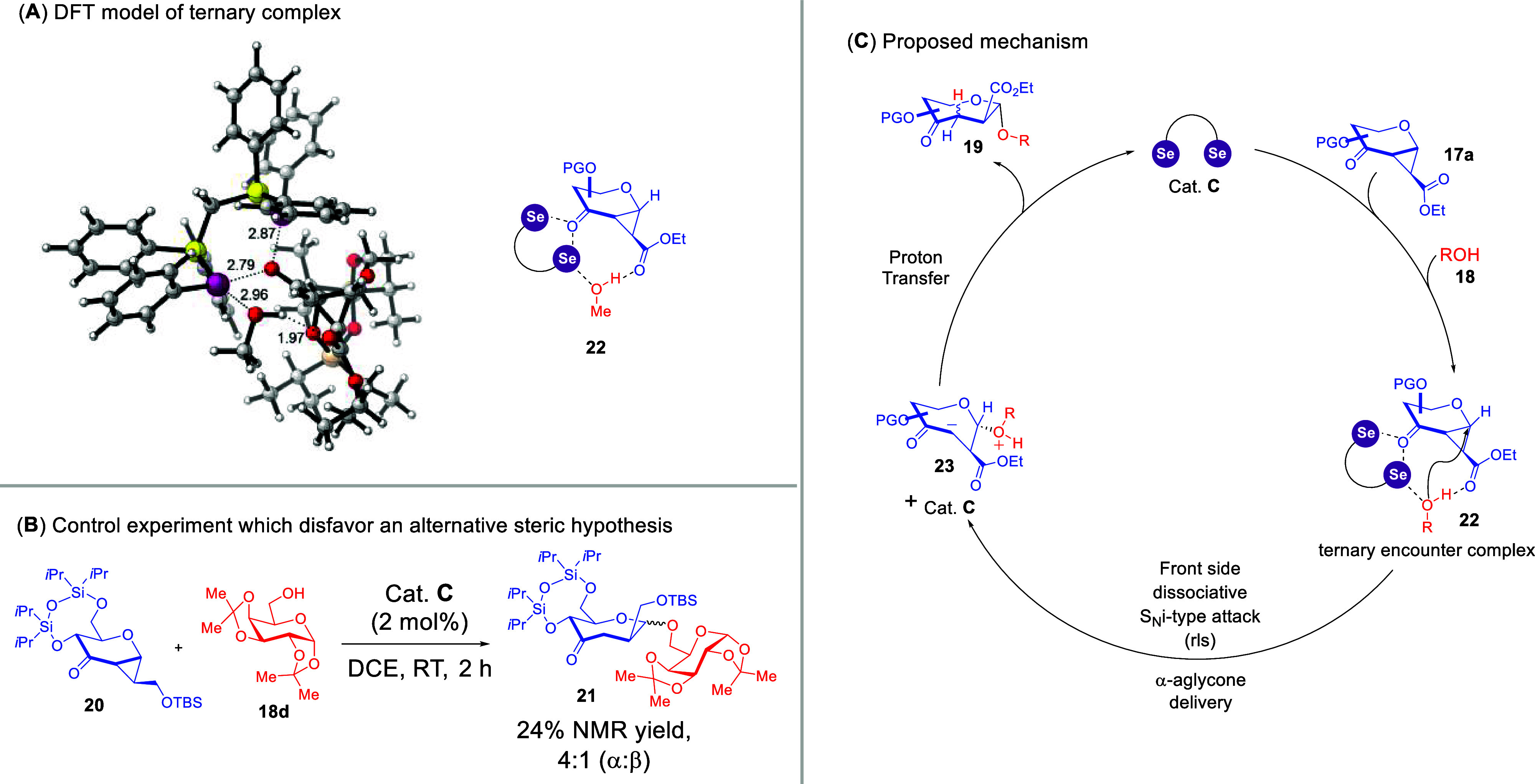
Computational and Experimental Studies That Contributed to the Mechanistic Proposal of the Septanosylation^a^

a Adapted from ref 2, under an open-access CC-BY 4.0 License, Copyright 2023, American Chemical Society, Washington, DC.

We also conducted control experiments by substituting the ester on the donor with a sterically bulky OTBS group in the control substrate **20**. The diminishment of anomeric selectivity (Scheme 9B) supports our postulate that the consistent α-selectivity we observed is due to noncovalent rather than steric influences. Furthermore, competitive ^13^C kinetic isotopic effect (KIE) experiments at natural abundance resulted in a reproducible value of 1.005. These data, along with positive kinetic orders, with respect to all substrates and catalyst led to the conclusion that a rare dissociative S_N_i (internal nucleophilic substitution) type mechanism^53^ is operative. We thus propose the following working hypothesis: a ternary donor–acceptor-catalyst noncovalent complex **22** was instrumental in guiding the aglycone delivery at the α-face to enforce the excellent α-selectivity in a front face S_N_i mechanism, which constitutes the rate-limiting step (rls). This is followed by a proton transfer, which eventually yielded the target *O*,*S*-septanosides **19**.

In light of increasing attention toward accessing hydrolytically stable *C*-glycosides,^54–56^ we further explored the capability of ChB catalysis to gain entry into *C*-septanosides. We were somewhat surprised that rare early efforts by Hoberg in using strained cyclopropanated donors^38,45^ for *C*-septanosylations resulted in unsatisfactory stereoselectivity outcomes.^49,50^ In a push to address these unresolved stereoselectivity challenges, we started our exploration of strain release *C*-septanosylation of **17**, using easily available silylated nucleophiles. By using catalyst **C** or **D**,^25^ a broad array of silylated nucleophiles **23** can be accommodated to yield the *C*-septanosides **24** with consistently excellent α-selectivity (Scheme 10).^57^

**Scheme 10:**
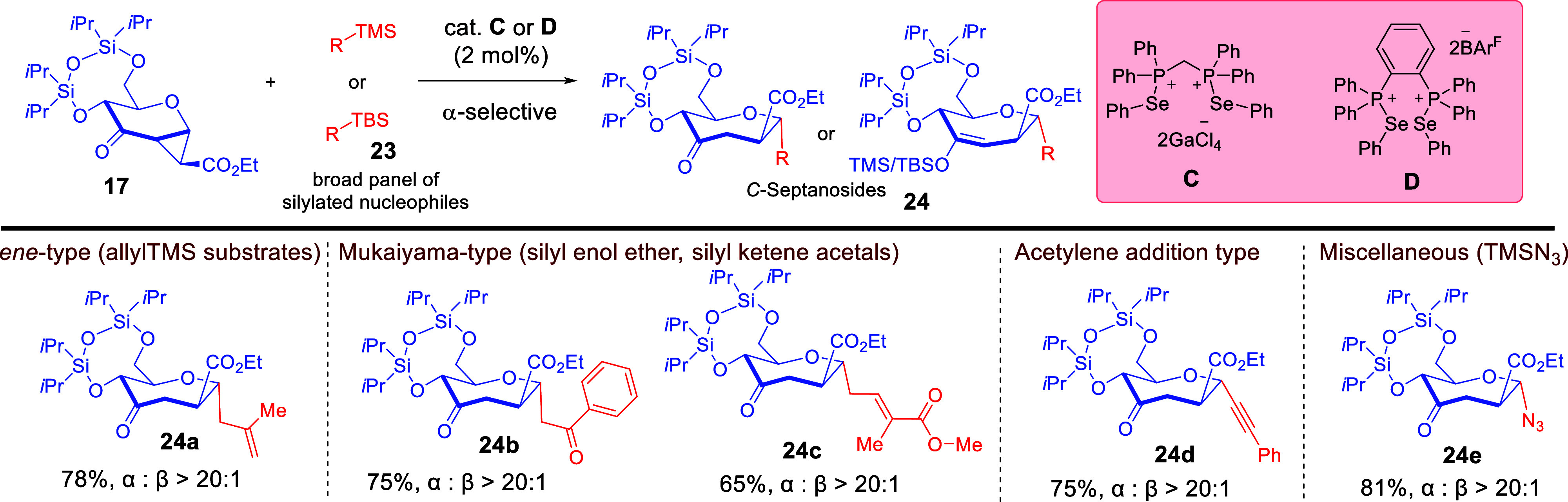
ChB-Catalyzed *C*- and *N*-Septanosylation Using Silylated Nucleophiles^a^

a Adapted from ref 57, under an open-access CC-BY-NC-ND 4.0 License. Copyright 2023, Wiley–VCH.

When we subjected the OTBS substrate **20** to the ChB-catalyzed *C*-septanosylation conditions, we unexpectedly found out that the ester to silyl ether substrate modification retained excellent anomeric selectivity (Scheme 11A). This hinted that a mechanistic shift occurred. Furthermore, the employment of a fully truncated cyclopropanated donor resulted in sterically dependent stereoselectivity diminishment over a range of different nucleophiles. Furthermore, we noticed the cruciality of the concurrent presence of the glycosyl donor and the catalyst in several sequential control experiments, while prestirring the catalyst with the silyl nucleophile was not viable. This suggested that a preferential formation of a ternary assembly comprising of substrates and catalyst over decomposition pathways could be vital in the catalyst activation stage. Accordingly, we propose the formation of ternary complex **26** where a blend of Se**···**O and Se**···**π interactions imparted by **C** concurrently activates the ketone and the olefin in the substrates (Scheme 11B). This is followed by either a silyl shuttling process involving Si–Se bond formation in **27**, or a stepwise ring expansion to access a pentacoordinate silicon intermediate **29** through enolate **28**. Finally, we propose that a substrate controlled intramolecular aglycone transposition process is in operation where α-selectivity is steered by the steric hindrance of the β-facing C2 ester moiety.

**Scheme 11:**
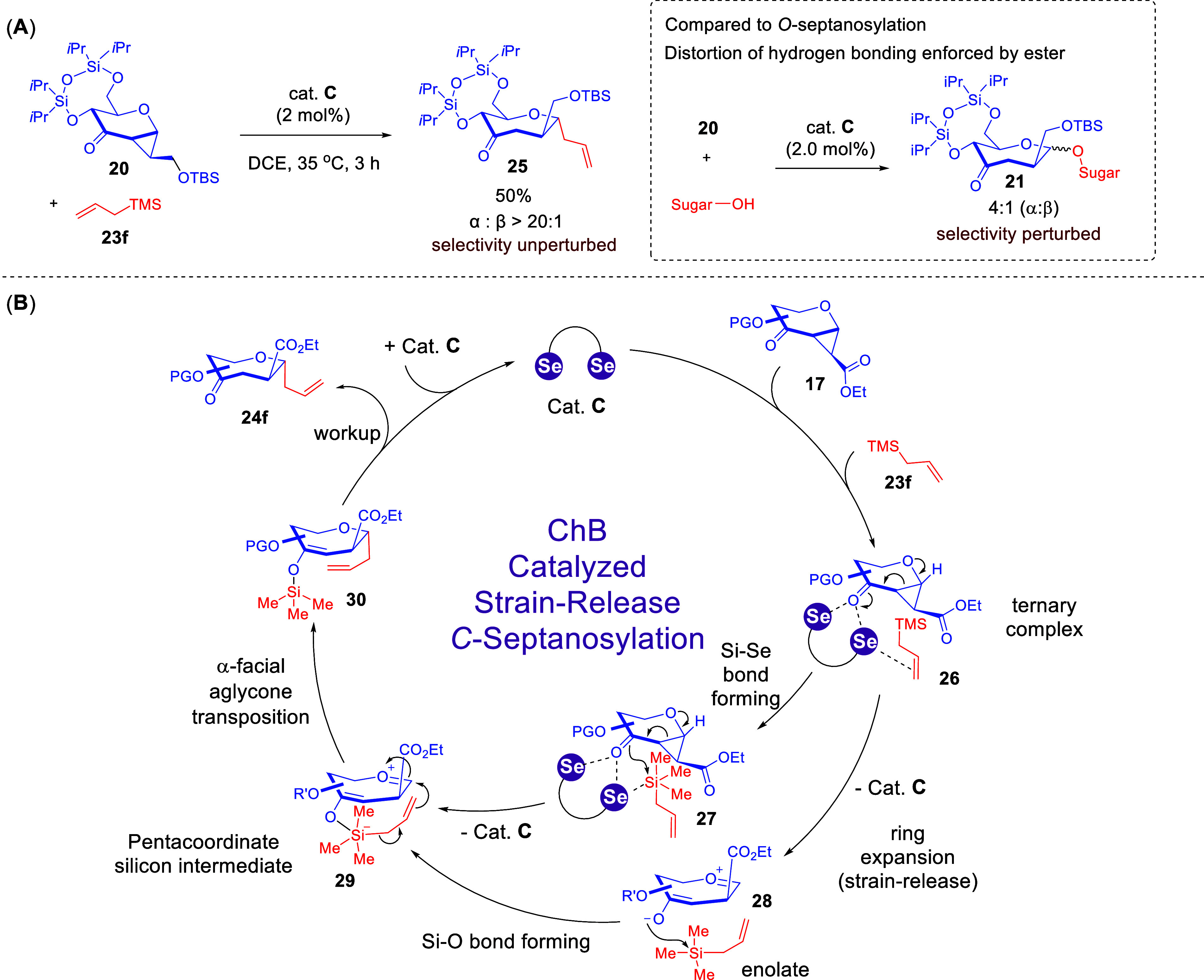
Mechanistic Switch to an Aglycone Transposition Using Silylated Nucleophiles^a^

a Adapted from ref 57, under an open-access CC-BY-NC-ND 4.0 License. Copyright 2023, Wiley–VCH.

Furthermore, we were interested to investigate novel routes into the challenging β-indolyl-glycoside scaffold due to their known biological relevance such as anti-inflammatory^58^ and antidiabetic activity.^59^ By investigating a palette of C2-substituted as well as *N*-substituted indoles as nucleophiles, we discovered that a facile *C*-2-deoxyglycosylation of glycals can be realized by using a Xanthphos-derived PCH catalyst **E** (Scheme 12).^60^ We further questioned whether the relatively rarer N1 nucleophilic property of indoles could also be tapped in a similar strategy to access the *N*-indolylglycoside analogues. Intriguingly, we discovered that a range of different C3-substituted, or C3,C2-annulated indoles can be successfully employed to gain entry into useful β-*N*-indolylglycosides **34**. A modification of the solvent system between toluene, CH_2_Cl_2_,, or a solvent mixture of both was necessary to obtain optimal results across different substrates.

**Scheme 12:**
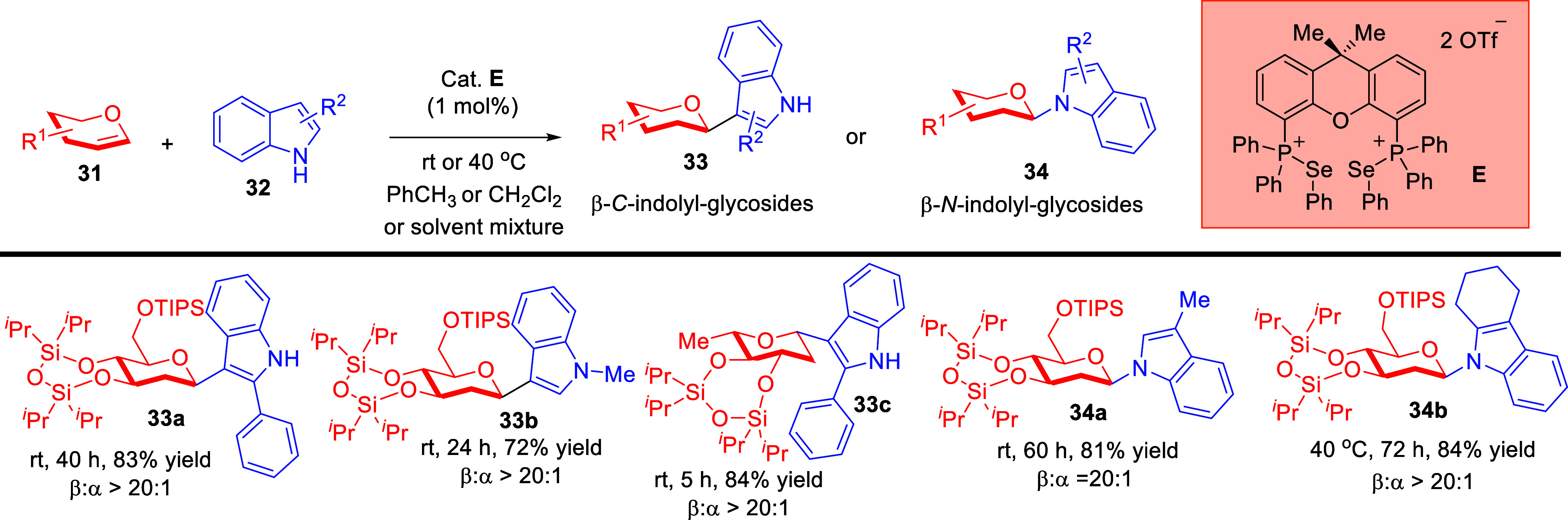
ChB-Catalyzed *C*,*N*-Indolyl Glycosylation with Glycals^a^

a Adapted from ref 60, under an open-access CC-BY-NC-ND 4.0 License. Copyright 2024, Wiley–VCH.

We further sought to understand the mechanistic intricacies of this strategy through ^1^H and ^77^Se NMR titrations (Scheme 13A). Besides observing downfield shifts in ^77^Se NMR, we were pleasantly surprised to note that substantial chemical shift perturbations were observed in the Se-Ph region of the ^1^H NMR spectra. This suggests that the aromatic flanks of **E** additionally participated in the catalysis. DFT modeling of the catalyst-glycal complex revealed an unexpected suite of Se**···**O and π-interactions that is distorting the^4^ *H*_5_ conformation of glycals into a *B*_*3,0*_ conformation (Scheme 13B). Such conformational distortions of glycosyl donors are known in enzymatic glycosylations,^15,61^ which can prepare glycosyl donors electronically and structurally toward nucleophilic attacks.

**Scheme 13:**
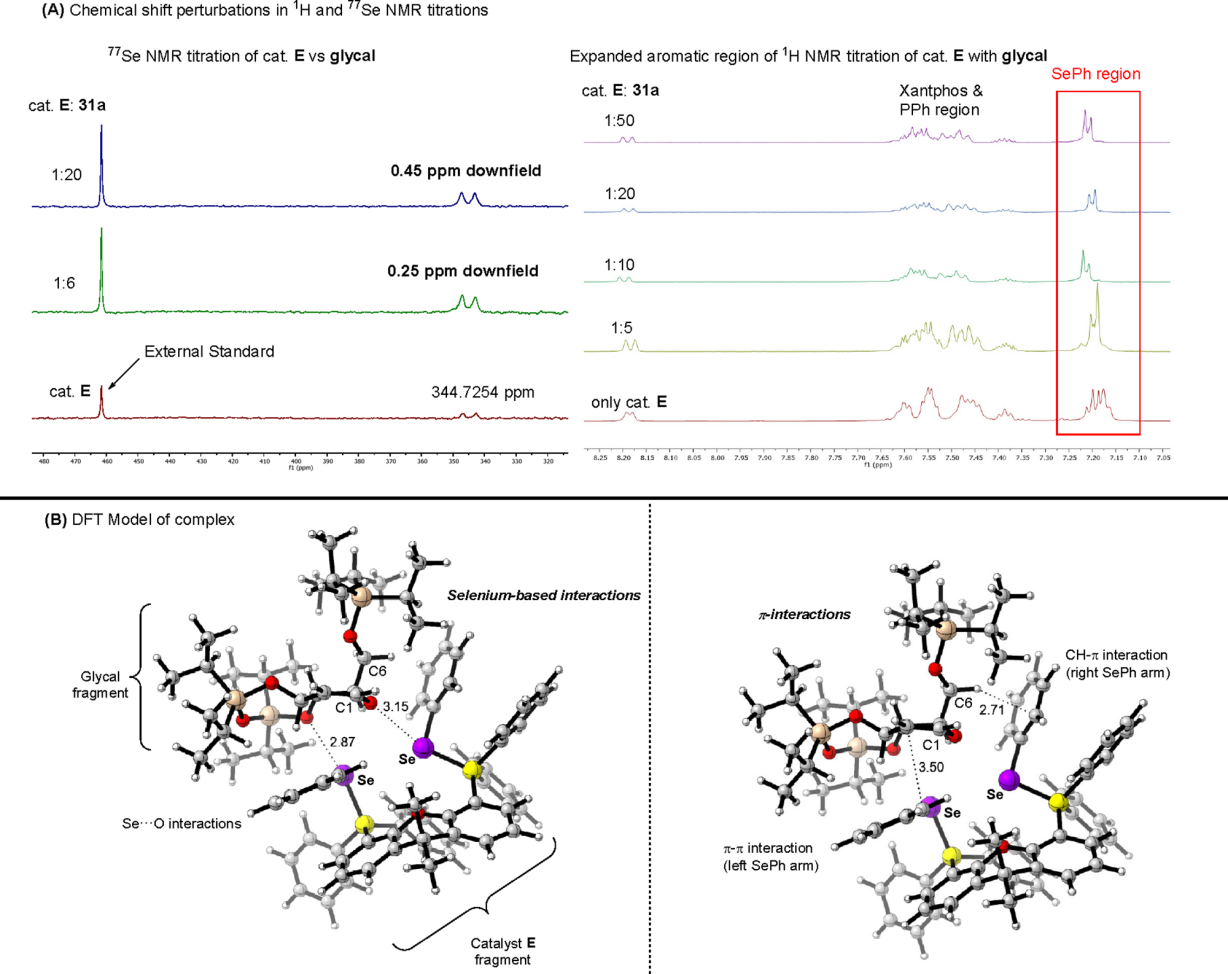
NMR Titration and DFT Modeling That Evidence Glycal Conformational Distortion^a^

a Adapted from ref 60, under an open-access CC-BY-NC-ND 4.0 License. Copyright 2024, Wiley–VCH.

We proposed a remarkable ChB-activation mechanism that first involved half-chair to boat conformational distortion of the glycal that is enforced by Se**···**O and π interactions imparted by the PCH catalyst (Scheme 14). This prepares a convex β-face that is relatively unobstructed for β-nucleophilic attack. In light of deuterated experiments, we postulated a subsequent C–C bond forming step on indole C3, followed by a series of water mediated proton transfers to form the final β-*C*-indolyl-glycoside **33**.

**Scheme 14:**
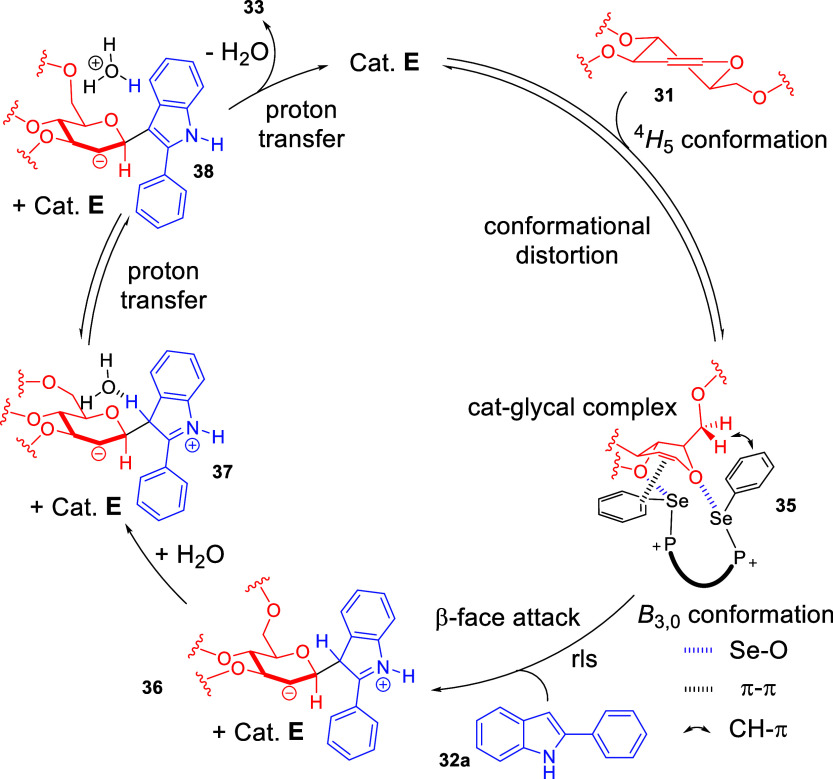
Proposed Mechanism for the ChB-Catalyzed 2-Deoxyglycosylation of Glycals with Indoles^a^

a Adapted from ref 60, under an open-access CC-BY-NC-ND 4.0 License. Copyright 2024, Wiley–VCH.

Additionally, we were keen to study if ChB catalysis could be employed in the stereoselective synthesis of glycomimetics, such as iminosugars,^62^ which are well-known to possess broad biological activity profiles.^63^ It is worthwhile to emphasize that, compared to standard glycosylations, catalytic iminoglycosylation methods are rare. Furthermore, known Brønsted-acid-catalyzed methods generally suffer from harsher conditions, require meticulously controlled anhydrous conditions, and possess limited substrate tolerance.^64^ Hence, we reasoned that the general mildness of reversible σ-hole activation manifolds could contribute substantially to synthesize biologically interesting sp^2^-iminosugars.^63,65^

We were excited to discover that catalyst **D** was capable to facilitate biologically interesting *O*-iminoglycosylations starting from sp^2^-iminoglycals **39** (Scheme 15).^66^ A remarkable attribute was the water tolerance of this strategy, which enabled the reaction execution at ambient conditions without rigorous water exclusion.^64^ Furthermore, we determined that the substrate scope was broad and encompasses alcohol nucleophiles with varying steric hindrances, it opens up access to the Tn antigen mimetic scaffold **41b**,^65^ tolerates thiols, and even accommodates less reactive iminoglycals with disarming protecting groups as in **41d**.

**Scheme 15:**
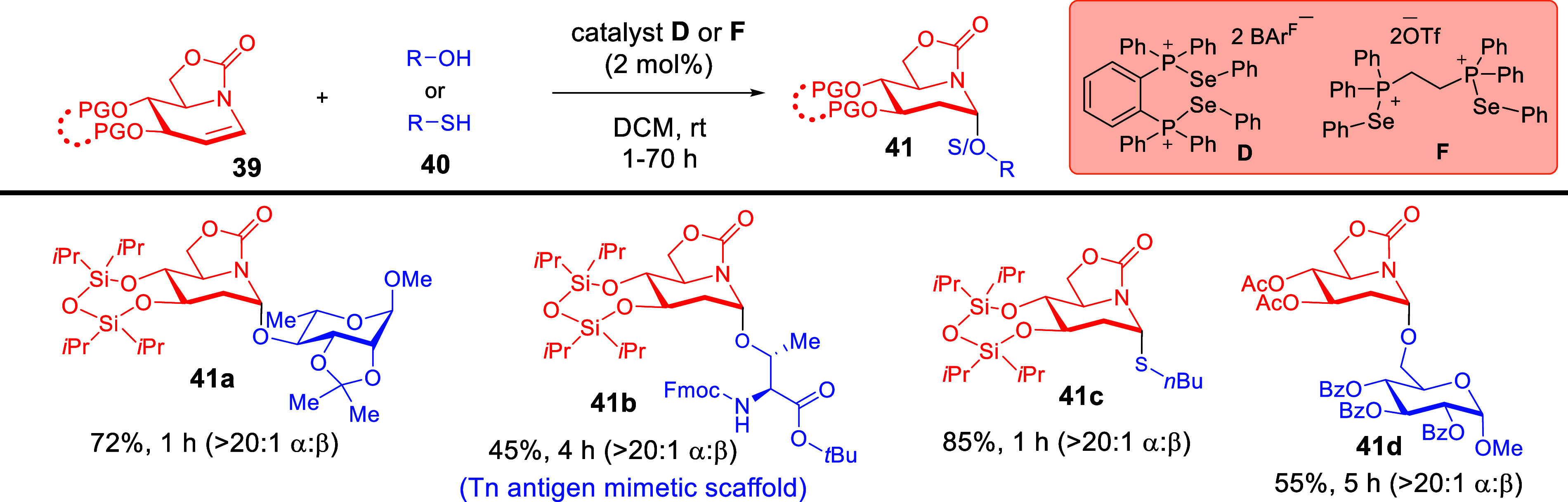
Facile ChB-Catalyzed Iminoglycosylation of Iminoglycals^a^

a Adapted from ref 66, under an open-access CC-BY 4.0 License. Copyright 2024, American Chemical Society, Washington, DC.

Mechanistically, we found that our ChB-catalyzed strategy deviated from classical 2-deoxyglycosylations. It is often presumed in the literature that a C2 protonation would first occur followed by nucleophilic attack on the oxacarbenium ion at the anomeric center.^41,43^ In contrast, ^1^H NMR titrations under ambient conditions revealed an unexpected water addition product **42** (Scheme 16A). Furthermore, subjecting **42** to the optimized catalytic conditions smoothly gave **41e** with excellent yields and anomeric selectivity (Scheme 16B). This downstream reaction can, however, be poisoned by a phosphine additive (Scheme 16C). This suggested that ChB-catalytic activation is vital in multiple elementary steps—an unexpected pathway that was not yet proven at that time.

**Scheme 16:**
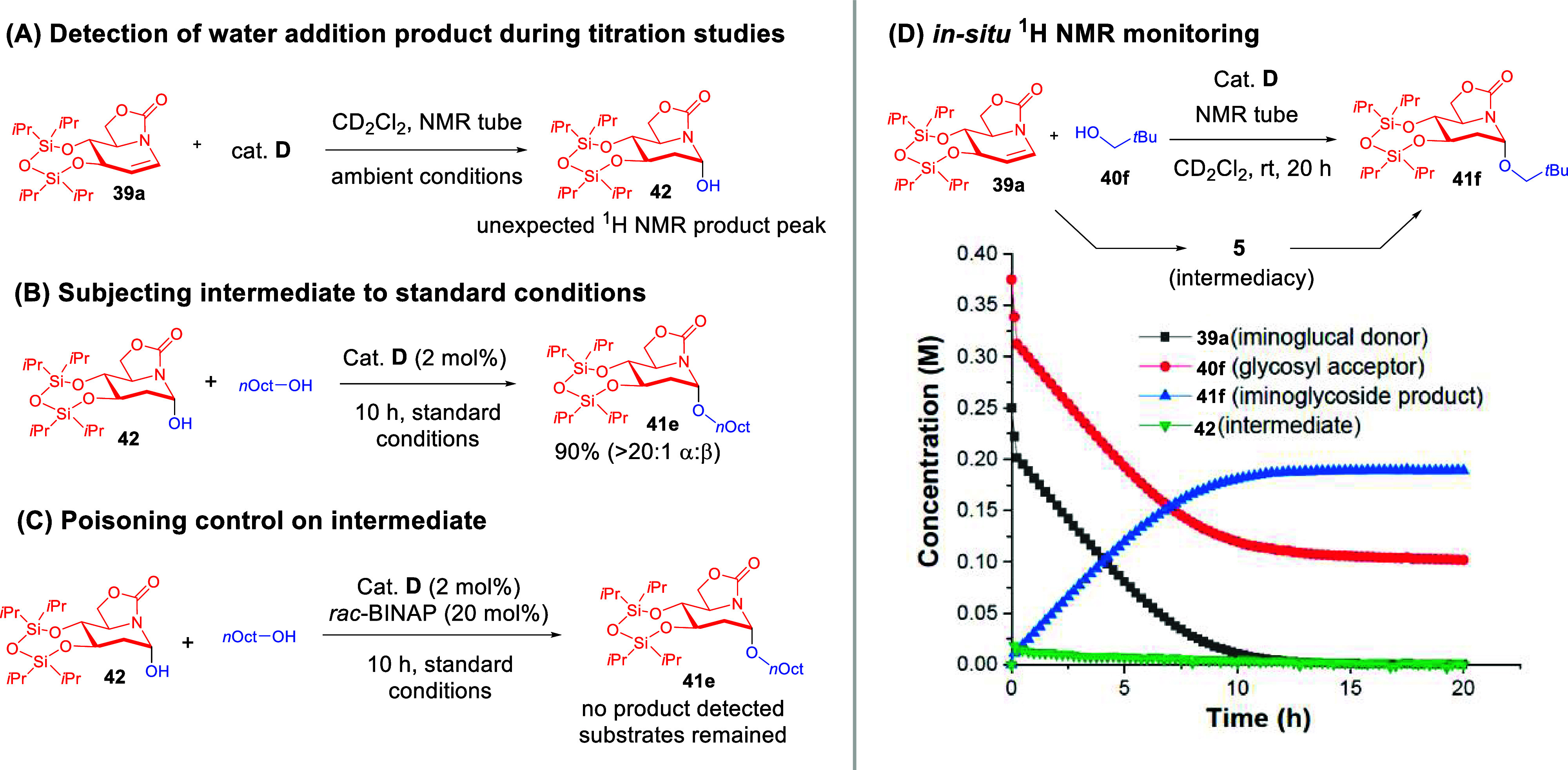
Experiments That Led to the Hypothesis of Multielementary Step ChB Activation^a^

a Adapted from ref 66, under an open-access CC-BY 4.0 License. Copyright 2024, American Chemical Society, Washington, DC.

We also detected the diminishing presence of water addition intermediate in the in-situ ^1^H NMR monitoring experiment (Scheme 16D), which aligns with the kinetic profile of a participating intermediate. Kinetic studies further revealed positive orders in glycosyl donor and catalyst and negative order in glycosyl acceptor. Along with DFT optimization that supported the plausibility of ChB-engagement modes of the catalyst with the glycal substrate, as well as with downstream intermediates (Scheme 17), we proposed the following multistep ChB-activation mechanism. The unprecedented mechanism (Scheme 18) is initiated with a PCH catalyzed addition of catalytic water to the iminoglycal’s olefin and then catalyst disengagement. This would generate **42**, which re-engages the ChB-catalyst downstream to facilitate a proton transfer in **44** to form a facile H_2_O^+^ leaving group on **45**. Following the departure of H_2_O and the formation of an iminoglycosyl cation **46**, the catalyst **D** engages in a bidentate activation mode, which facilitates the final iminoglycosylation step.

**Scheme 17:**
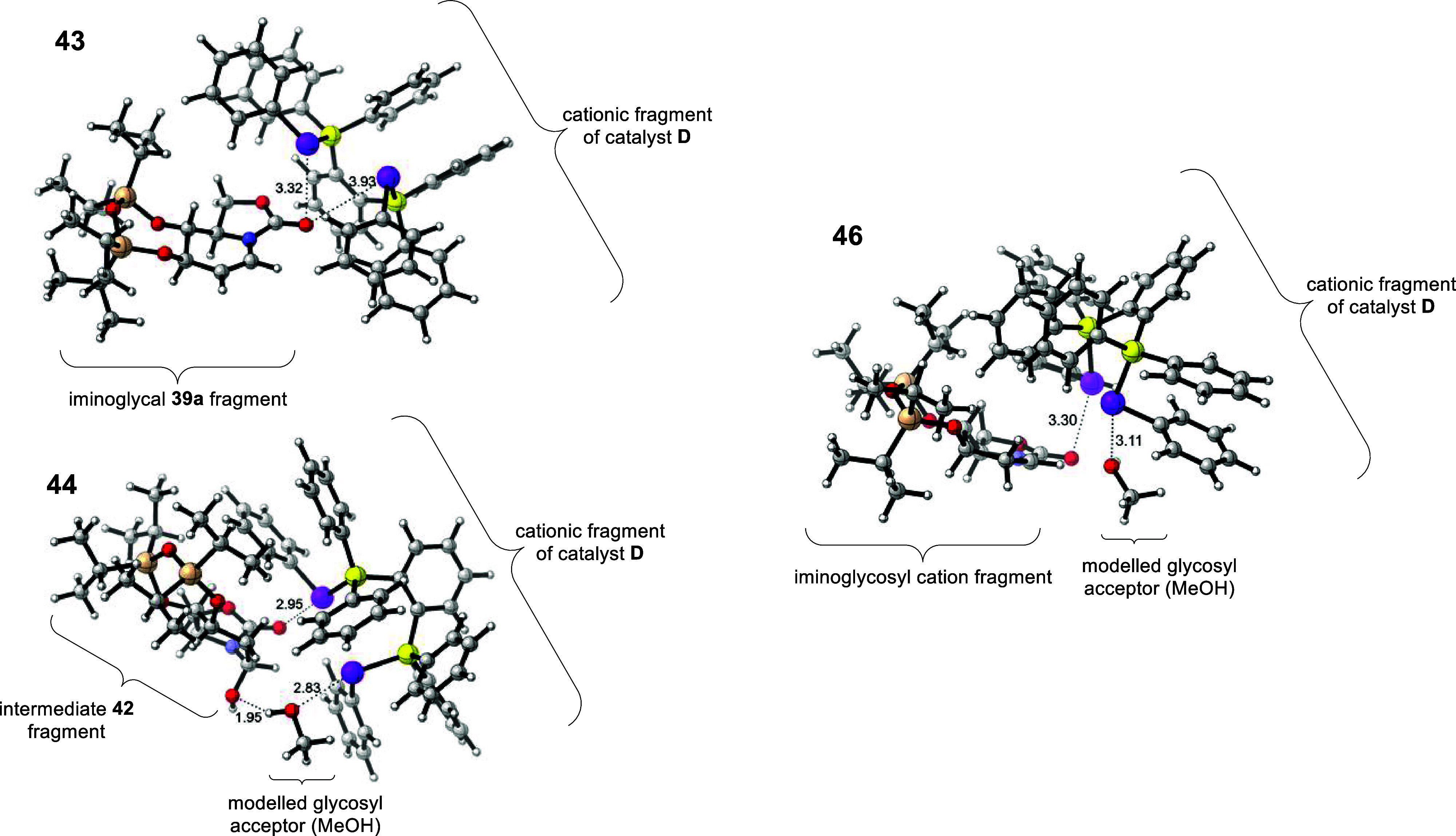
DFT-Computed Intermediates That Are Proposed in the ChB-Catalyzed Iminoglycosylation^a^

a Adapted from ref 66, under an open-access CC-BY 4.0 License. Copyright 2024, American Chemical Society, Washington, DC.

**Scheme 18:**
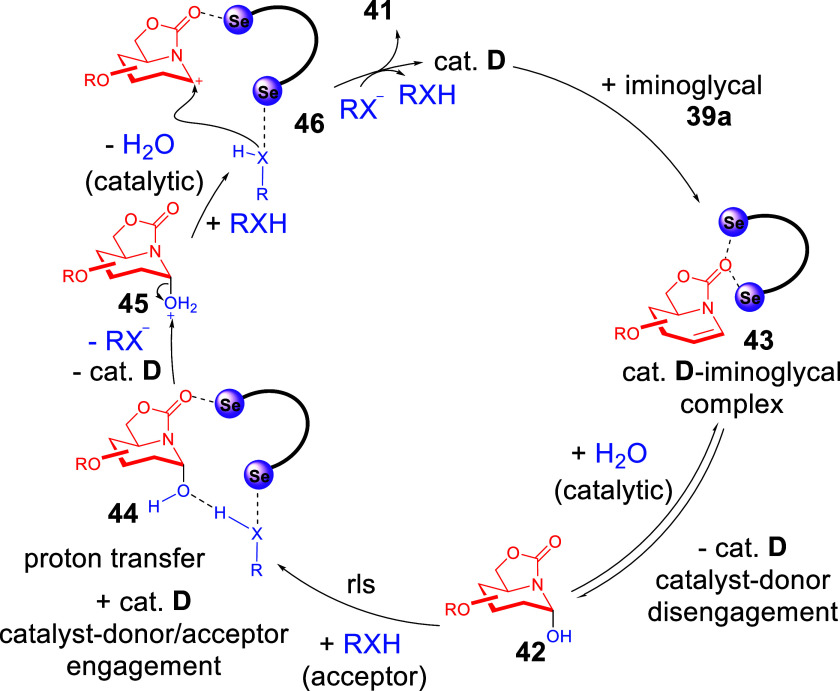
Proposed Mechanism of Multistep ChB-Catalyzed Iminoglycosylation^a^

a Adapted from ref 66, under an open-access CC-BY 4.0 License. Copyright 2024, American Chemical Society, Washington, DC.

## 3 EXPLOITATION OF ASYMMETRIC CATALYSIS TO SIMULTANEOUSLY TACKLE MULTIPLE STEREOCHEMICAL DIMENSIONS IN SITE-SELECTIVE CARBOHYDRATE FUNCTIONALIZATIONS

Another parallel strategy that we are interested in is to open new complexity generation routes to access unnatural glycosidic chemical space by harnessing the chiral information within an asymmetric catalytic system.^11,14^ We have made progress in this domain by identifying a suite of asymmetric rhodium, palladium, and copper catalytic systems that can concomitantly surmount the host of site-,^8–11,32^ diastereo-, enantio-, and dynamic kinetic resolution^35^ challenges that are endowed in such transformations. Significantly, while we noted that our observed 3-OH functionalizations can be partially attributed to a substrate-controlled bias, we emphasize the cruciality of chiral catalyst control in our strategies, as the use of the correct enantiomer of the ligand was critical in achieving the excellent regioselectivity across the broad array of carbohydrate polyols.

### 3.1 Synergistic Chiral Rh­(I) and Organoboron Catalysis for Desymmetrizative Functionalizations of Carbohydrate Polyols^3^

In light of the scarce availability of asymmetric catalytic reports in 2022 that were amenable to the use of prochiral electrophiles on carbohydrate polyols, we embarked on a Rh­(I)-catalyzed oxanorbornadiene desymmetrization strategy^31,67^ that could potentially pave unprecedented routes into biologically relevant arylhydronapthalene glycosides^68^ (Scheme 19).^3^ Notably, direct stereoselective access into these scaffolds was previously not available.

**Scheme 19:**
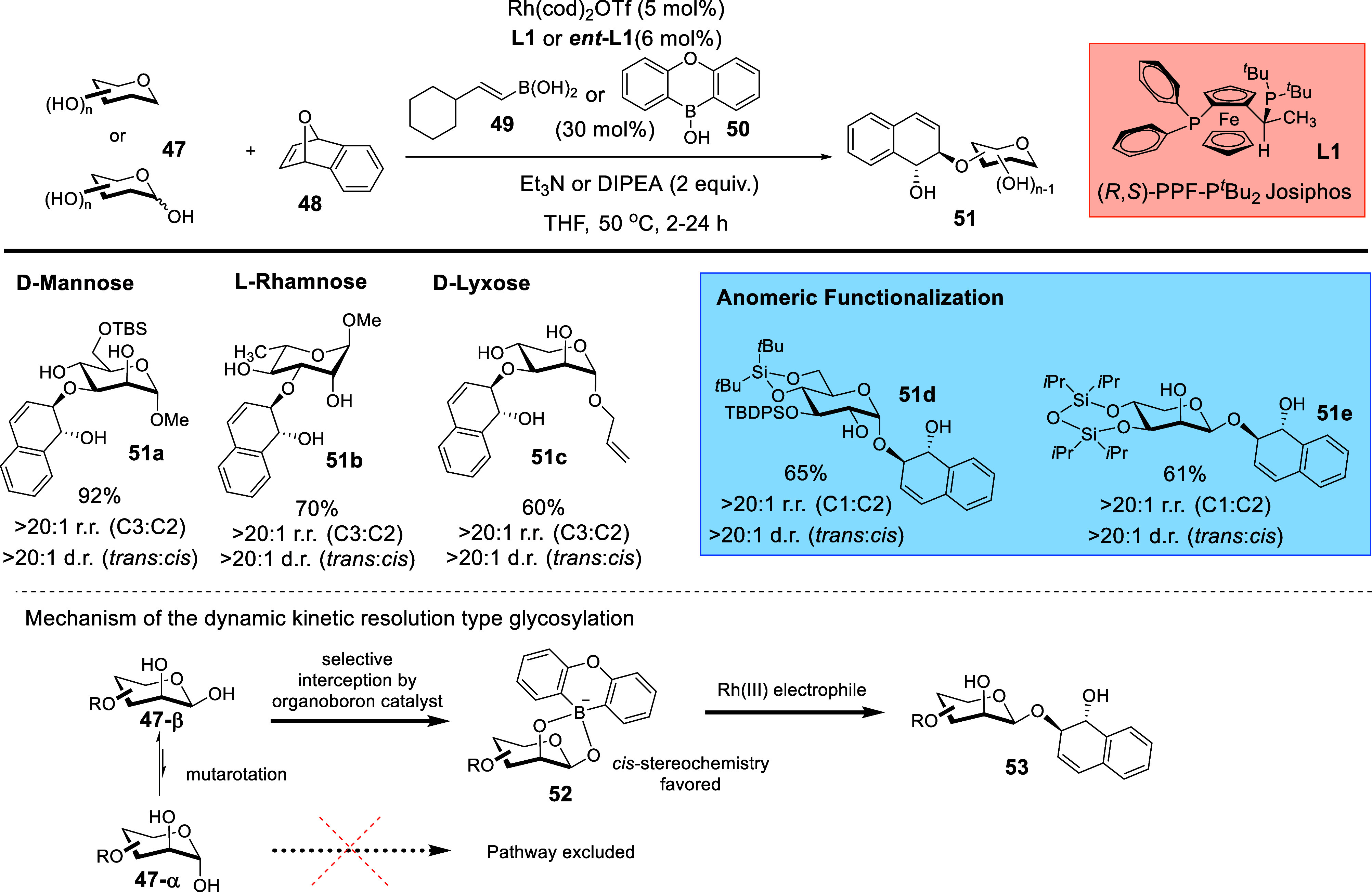
Synergistic Chiral Rh­(I)/Organoboron Catalyzed Site-Selective Functionalization of Carbohydrate Polyols to Access Arylhydronapthalene Glycosides^a^

a Adapted and modified from ref 3, under an open-access CC-BY 4.0 License. Copyright 2023, Springer Nature.

After evaluation of a panel of chiral ligands, we discovered that the Rh­(I)/(*R*,*S*)-PPF-P^*t*^Bu_2_ Josiphos (**L1**) asymmetric system, in conjunction with a judiciously selected organoboron catalyst,^69^ offered a robust substrate scope. In addition to excellent site-selectivity control over a broad range of monosaccharides to access **51a**–**51c**, we noted that rarely observed dynamic kinetic resolution-type glycosylation on anomeric unprotected reducing sugars proceeded smoothly. This afforded entry into highly demanded 1,2-*cis*-glycosides such as the α-1,2-*cis*-glucosides and the β-1,2-*cis*-lyxosides **51d** and **51e**. This achievement would additionally require the synergistic catalytic system to intercept one of the two rapidly equilibrating anomers **47-β**, **47-α** at the hemiacetal moiety (Scheme 19), and thereafter funneling the catalytic process selectively toward one specific anomer **53**. It is also vital to mention that when the opposite enantiomer of the ligand was employed, the mismatching combination resulted in diminishment of regioselectivity over a range of sugars such as mannose, galactal, arabinose, lyxose, and 1,6-anhydromannose based substrates.

It is important to emphasize the chiral catalyst-controlled facet of this strategy, as a control experiment that utilized the achiral congener 1,1′-bis­(diphenylphosphino)­ferrocene (dppf) gave hugely diminished site-selectivity. We also determined through NMR kinetic studies that the reaction was first order, with respect to both rhodium and the organoboron catalyst. As such, we proposed two intersecting catalytic cycles that involved reversible covalent complexation of the organoboron catalyst **49** with a diol motif on **47a**, as well as a Rh­(I)/Rh­(III) couple after oxidative addition to **48** (Scheme 20). Both catalytic cycles would then converge at the borinate **58** and the Rh­(III) **56** resting states, which ultimately sets the stage for a precise C–O functionalization that involves multiple stereocontrol to generate the desired arylhydronapthlene glycosides **51**.

**Scheme 20:**
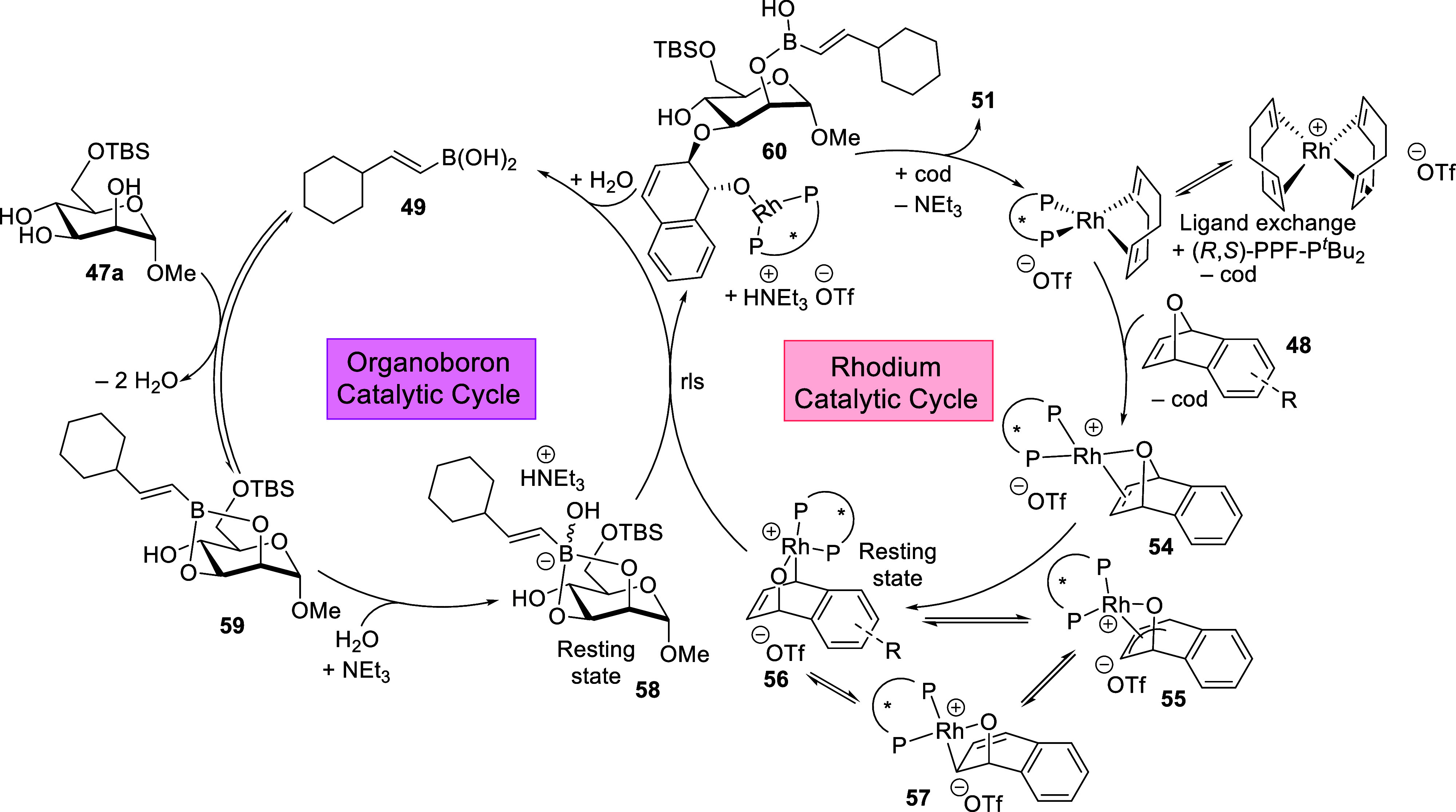
Working Mechanistic Hypothesis of the Synergistic Rh­(I)/Organoboron Catalysis^a^

a Adapted from ref 3, under an open-access CC-BY 4.0 License. Copyright 2023, Springer Nature.

### 3.2 Synergistic Chiral Pd and Organoboron Catalysis That Involved Noncovalent Stereocontrol

The employment of Pd/organoboron catalysis in the Tsuji–Trost-type allylation of carbohydrate polyols was previously reported by Niu and co-workers using achiral ligands;^70^ however, the concomitant generation of an exogenous chiral center was not feasible. Lately, we reported an interesting instance of a synergistic asymmetric Pd/organoboron-catalyzed site-selective functionalization of carbohydrate polyols **61** with alkoxyallenes **62** (Scheme 21),^4^ where the themes of NCIs and asymmetric transition-metal catalysis intersect. The chiral DACH-naphthyl Trost ligand **L2** employed in our strategy was classically recognized to impart stereoselectivity through steric influences. However, early optimizations revealed that solvents that could potentially forge HBs with saccharides such THF, 1,4-dioxane and acetonitrile gave diminished site-selectivity and diastereoselectivity, while less polar solvents like toluene or dichloromethane gave excellent selectivity. This intriguing observation led us to question whether NCIs could be operating in the stereoselectivity controlling step.

**Scheme 21:**
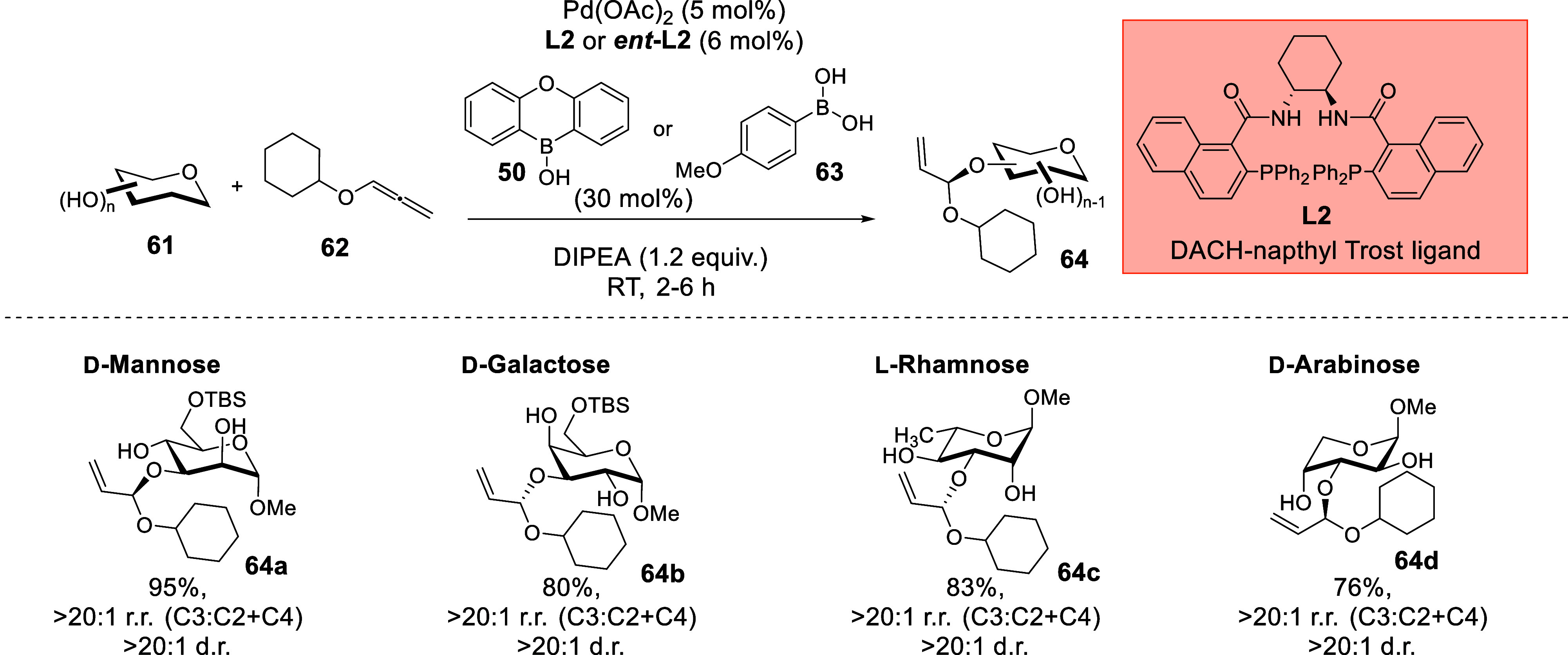
Synergistic Pd/Organoboron-Catalyzed Site-Selective Functionalization of Carbohydrate Polyols with Alkoxyallenes.^4^ ^a^

a Adapted from ref 4, under an open-access CC-BY-NC-ND 4.0 License. Copyright 2024, Wiley–VCH.

Further control experiments yielded deeper insights. Increasing the amounts of 1,4-dioxane additives to our standard optimized conditions resulted in the worsening of diastereoselectivity and site-selectivity (Scheme 22A). Furthermore, it was also surprising that 4-OH PMB (*p*-methoxybenezene)-substituted mannosides **65** led to diminished site-selectivity (Scheme 22B). This further hinted that 4-OH could be contributing vital HB in the stereoselectivity controlling step.

**Scheme 22:**
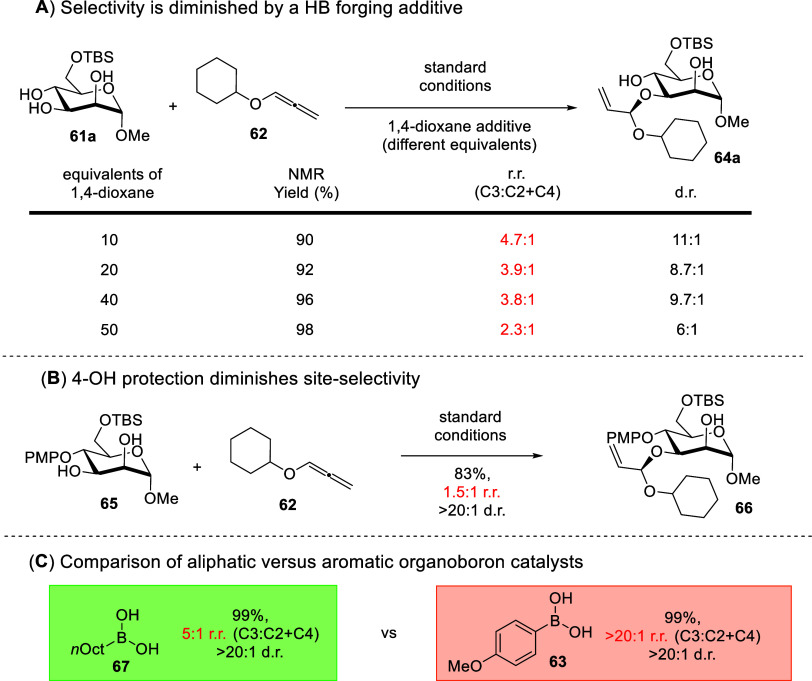
Experiments That Evidence the Participation of HB and CH−π Interactions in the Stereocontrol^a^

a Adapted from ref 4. under the open-access CC-BY-NC-ND 4.0 License. Copyright 2024, Wiley–VCH.

On the other hand, we also noted that organoboron catalysts with aromatic substituents yielded a much better selectivity, compared to those with aliphatic scaffolds (Scheme 22C). A comparison of the individually measured ^1^H NMR spectra of the Pd-π-allyl complex and the borinate resting state catalyst with those of the encounter complex revealed upfield shifts in the ether and allylic protons, likely due to the anisotropic effect. Furthermore, the aromatic protons displayed a downfield chemical shift perturbation. This series of experiments supports the postulate that CH−π interactions are plausibly contributing to the stereoselectivity control.

Benefiting from DFT modeling of the reaction path leading to the C3 functionalized product, we further noted a decrease of the hydrogen bonding distance along the reaction coordinate (Scheme 23A), which further strengthens the postulate of HB influence in the stereocontrolling step.^71^ We hence proposed the simultaneous operation of palladium and organoboron catalysis, which converges at the encounter complex **69** that comprises a suite of stereocontrolling HB and CH−π interactions (Scheme 23B). This blend of NCIs is vital in the subsequent stereoselectivity controlling step to enforce the 3-OH site-selectivity and chirality center creation to yield the target allylated saccharide **64**.

**Scheme 23:**
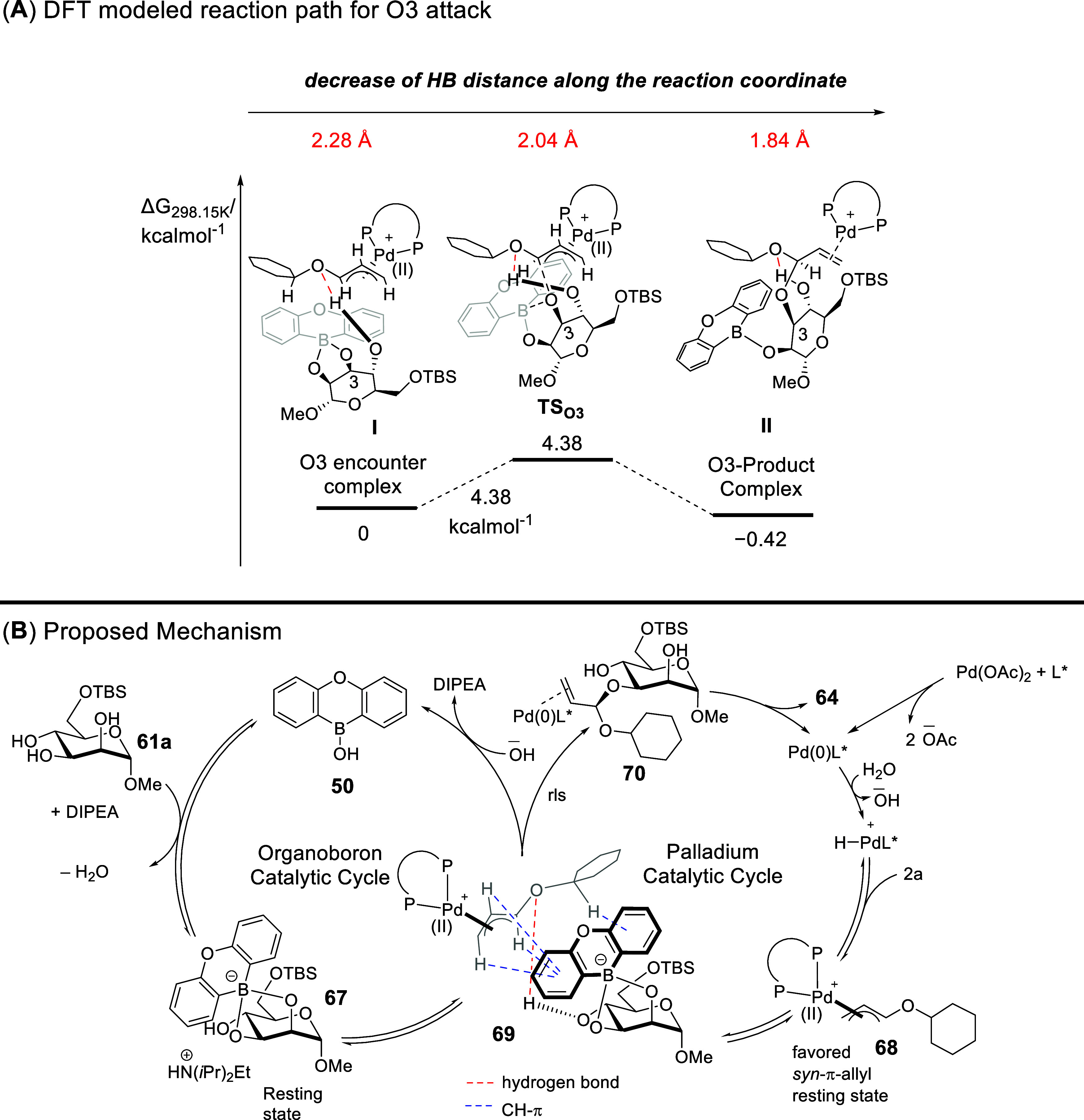
DFT-Modeled Reaction Path and Proposed Mechanism for the Pd/Organoboron Catalysis^a^

a Adapted from ref 4 under an open-access CC-BY-NC-ND 4.0 License. Copyright, 2024, Wiley–VCH.

### 3.3 Enantioconvergent and Site-Selective Chiral Cu Radical Catalysis for Precise Carboxamidation of Minimally Protected Saccharides

While the previous two strategies required organoboron reagents, the sole use of a single asymmetric catalyst to overcome the host of stereoselectivity challenges is rarer and demands a higher level of catalytic control. In light of recent intense interest of employing radical involved catalytic strategies in carbohydrate synthesis,^54–56^ we were interested to investigate into the unexplored domain of chiral radical catalytic control^72^ in carbohydrate chemistry.

Gaining design inspiration from the recent attention given to enantioconvergent radical catalytic strategies in C–O bond forming reactions,^74^ we were curious if such platforms could be further used to contemporaneously tackle the carbohydrate site-selectivity issue in etherifications. Our catalyst evaluation revealed that a Cu­(I) catalytic system coupled with an *i*Pr-*bis*-oxazolidine (BOX) ligand **L3** is optimal in tackling the site-selectivity and diastereoselectivity challenges over a broad series of naturally occurring sugar substrates (Scheme 24).^73^ The fact that no organoboron co-catalyst was required further substantiates the robustness of this protocol. Furthermore, dynamic kinetic resolution-type glycosylation was also amenable on reducing sugars, and β-anomers were consistently generated in **74a** and **74b**, regardless of the stereochemistry of the C2 substituent. This feature further suggests that 1,2-diol chelation by the copper catalyst is not operative.

**Scheme 24:**
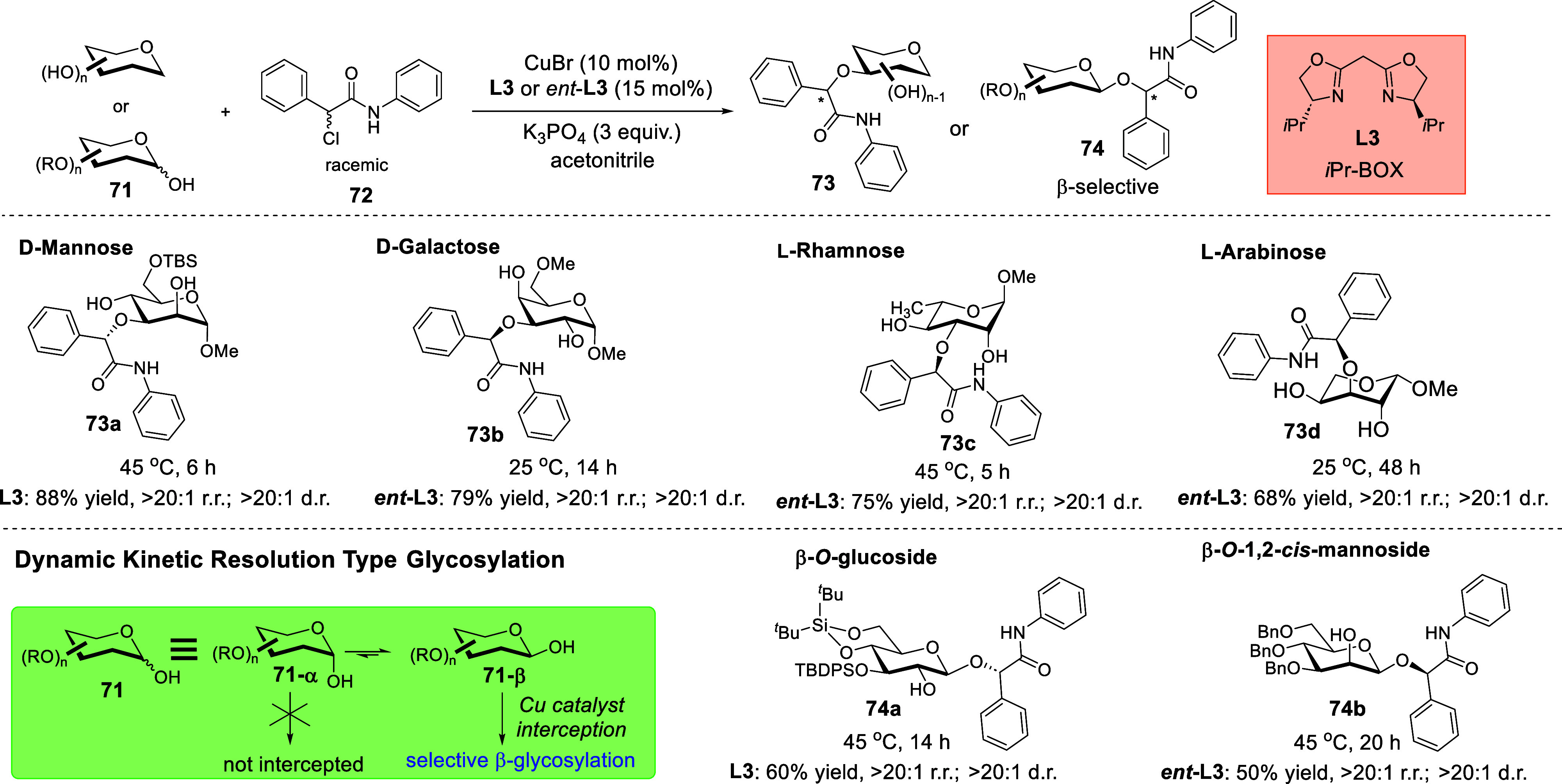
Site-Selective and Enantioconvergent Etherification through Chiral Copper Radical Catalysis^a^

a Adapted from ref 73, under an open-access CC-BY-NC-ND 4.0 License. Copyright 2024, Wiley–VCH.

TEMPO trapping and radical clock experiments further ascertained the radical involved nature of the chiral copper catalysis (see Schemes 25A and 25B). Guided by DFT calculations of the radical interception and reductive elimination steps, we propose a Cu­(I)/(II)/(III) catalytic cycle that involves single electron transfers (see Scheme 26). The mechanism starts with the displacement of the bromide on CuBr by a molecule of the acetonitrile solvent, and the bromide remains as a spectator ion in the mechanism. The subsequent key steps include an enantioconvergent formation of a *C*-centered radical **R^•^** that resulted in the generation of Cu­(II) species **80**. This is followed by a series of reversible elementary steps: (i) A base promoted regioselective deprotonation of the polyol, (ii) a ligand exchange to form the Cu­(II)–O bond in **81**, and (iii) an interception of the *C*-centered radical with the Cu­(II) intermediate to access the high valent Cu­(III) species **82**, which eventually culminates in a reductive elimination that controls several stereochemistry dimensions to form the final etherified sugar **73a**. The reversibility of these final elementary steps is essential for the catalytic system to arrive at the optimal matched combination prior to the C–O bond construction at the reductive elimination step.

**Scheme 25:**
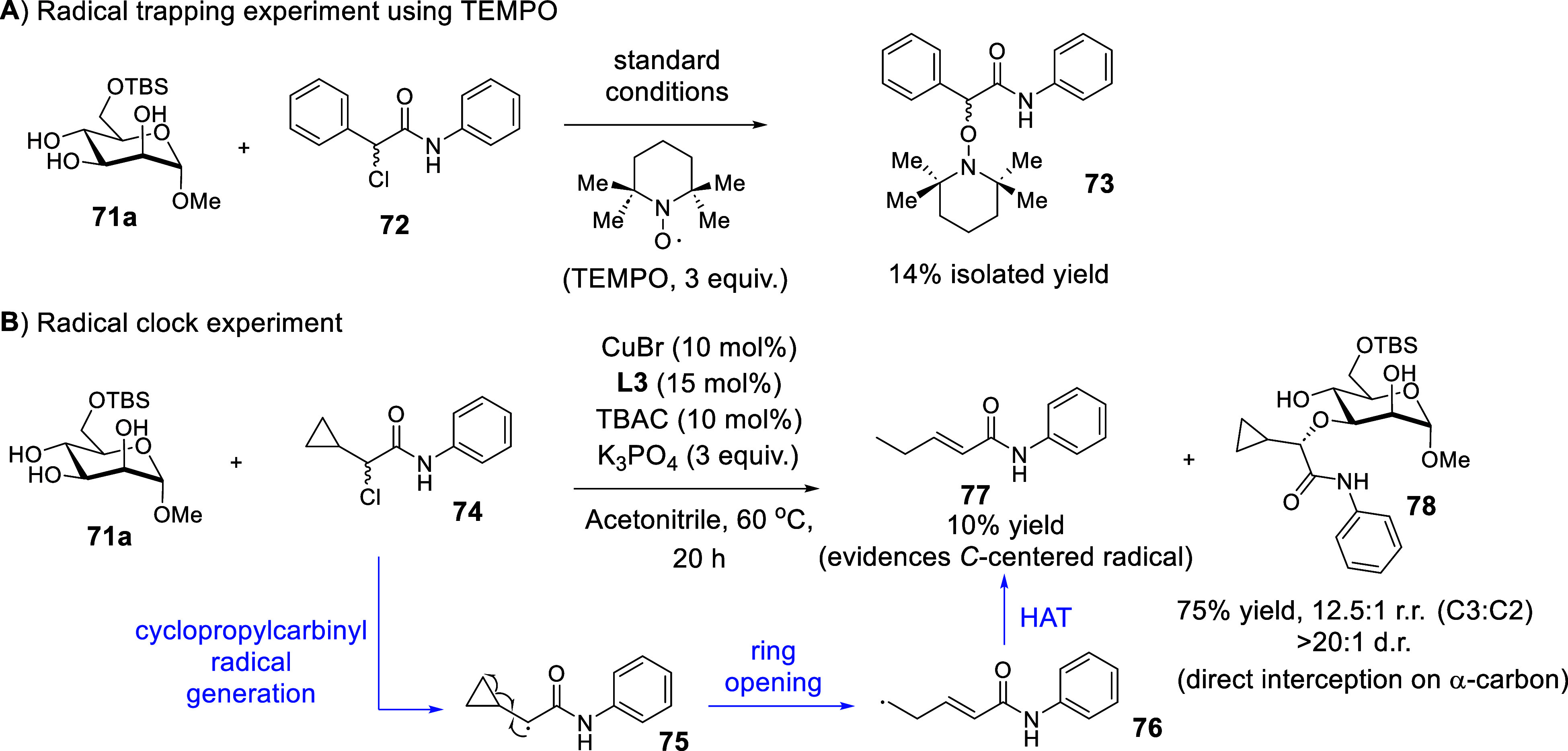
Radical Probe Control Experiments Evidence a Radical Pathway^a^

a Adapted from ref 4, under an open-access CC-BY-NC-ND 4.0 License. Copyright 2024, Wiley–VCH.

**Scheme 26:**
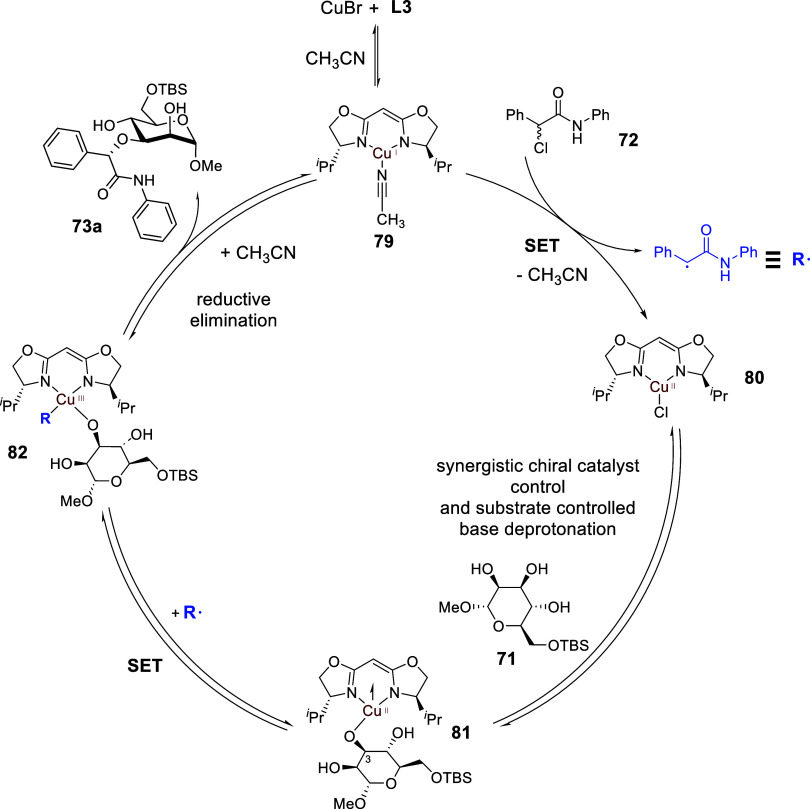
Site-Selective and Enantioconvergent Etherification through Chiral Copper Radical Catalysis^a^

a Adapted from ref 4, under an open-access CC-BY-NC-ND 4.0 License. Copyright 2024, Wiley–VCH.

## 4 SUMMARY AND OUTLOOK

In this Account, we provide a first-hand narrative of our group’s contributions to stereoselective carbohydrate synthesis by the debut of the σ-hole-based catalytic glycosylation strategy and by identifying chiral catalytic systems for site-selective carbohydrate functionalizations. Moving beyond field-specific glycosyl substrate-based approaches,^13,16^ our enriching journey unraveled hitherto undiscovered opportunities when the fields of supramolecular chemistry, asymmetric synthesis, and carbohydrate chemistry converge. Significantly, we brought about evidence that the mechanistic pathways of glycosylations were profoundly altered through the use of nonclassical σ-hole based interactions in catalysis, culminating in reactivity and stereoselectivity benefits. Furthermore, we identified strategies where a challenging blend of site-selectivity, diastereoselectivity, enantioselectivity, and anomeric selectivity hurdles in carbohydrate polyol functionalizations can be surmounted by asymmetric catalysis.

As a rapidly increasing body of researchers embraces interdisciplinary endeavors in unearthing innovative solutions, we believe that the true appreciation of stereoselectivity problems in the carbohydrate realm as fundamental synthetic problems is experiencing a broad renaissance among a wide spectrum of organic chemists.^12,75^ It is worthwhile to recall that historical core chemistry contributions to radical chemistry^76^ and stereoelectronic effects^77^ were indeed advanced by investigating carbohydrates. At this watershed juncture, we are optimistic that our generation is only at the cusp of the renewed realization that undiscovered gems still await at the interface of frontier synthetic concepts and stereoselective carbohydrate synthesis.
